# The Activity of *Chelidonium majus* L. Latex and Its Components on HPV Reveal Insights into the Antiviral Molecular Mechanism

**DOI:** 10.3390/ijms23169241

**Published:** 2022-08-17

**Authors:** Oskar Musidlak, Alicja Warowicka, Justyna Broniarczyk, Damian Adamczyk, Anna Goździcka-Józefiak, Robert Nawrot

**Affiliations:** Department of Molecular Virology, Institute of Experimental Biology, Faculty of Biology, Adam Mickiewicz University in Poznan, Uniwersytetu Poznanskiego 6, 61-614 Poznan, Poland

**Keywords:** *Chelidonium majus* L., greater celandine, defense-related proteins, alkaloids, human papillomavirus, HPV pseudovirions, plant latex, antiviral activity

## Abstract

Yellow-orange latex of *Chelidonium majus* L. has been used in folk medicine as a therapeutic agent against warts and other visible symptoms of human papillomavirus (HPV) infections for centuries. The observed antiviral and antitumor properties of *C. majus* latex are often attributed to alkaloids contained therein, but recent studies indicate that latex proteins may also play an important role in its pharmacological activities. Therefore, the aim of the study was to investigate the effect of the crude *C. majus* latex and its protein and alkaloid-rich fractions on different stages of the HPV replication cycle. The results showed that the latex components, such as alkaloids and proteins, decrease HPV infectivity and inhibit the expression of viral oncogenes (E6, E7) on mRNA and protein levels. However, the crude latex and its fractions do not affect the stability of structural proteins in HPV pseudovirions and they do not inhibit the virus from attaching to the cell surface. In addition, the protein fraction causes increased TNFα secretion, which may indicate the induction of an inflammatory response. These findings indicate that the antiviral properties of *C. majus* latex arise both from alkaloids and proteins contained therein, acting on different stages of the viral replication cycle.

## 1. Introduction

Plants are exposed to many dangers, especially from various pathogens living in their environment. For this reason, they have evolved complex defense mechanisms against pathogens and other stress factors, and thus, they can survive infections and cope with stressful conditions. Plants utilize a variety of approaches for their defense against pathogens, ranging from physical means such as thorns and bark to biochemical substances that have antimicrobial properties. The defense system of medicinal plants used in folk medicine is particularly well developed, especially in those species that produce latex, also known as milky sap. It is estimated that latex is produced by 12,500–20,000 plant species representing at least 20 phylogenetically unrelated families, such as Euphorbiaceae, Asteraceae, Apocynaceae, and Papaveraceae [1,2,3]. As a result, there are large discrepancies in the contents of proteins and low-molecular compounds between latices of different plant species. This example of convergent evolution and the fact that latices of many plants exhibit defense-related properties suggest that latex production contributes to plant resistance and survival [4,5]. Plant latex contains proteins and numerous low-molecular compounds, such as alkaloids, terpenoids, starch, oils, sugars, tannins, gums, carotenoids, organic acids, saponins, tannins, and phenols [3,5,6,7,8,9,10]. They are involved in a wide range of defense mechanisms, including those against viral infections. Activity against viruses infecting humans such as influenza virus, human papillomavirus, and others has been reported for various latex-bearing plant species [11,12,13]. Due to its healing properties, latex from plants has been used in traditional medicine to treat many diseases, and compounds isolated from the latex are used in conventional medicine. An example of such a latex-bearing plant that has long been used in European and Asian folk medicine is *Chelidonium majus* L., also known as greater celandine. It is a perennial which grows in the lowlands and foothills, on the edges of deciduous forests, and in parks. As a ruderal plant it also grows in built-up areas, gardens, and along fences, roads, and buildings. *C. majus* belongs to the family Papaveraceae and has long been used in European and Asian traditional medicine to remove skin lesions in the form of warts, papillae, and condylomas, which are visible symptoms of human papillomavirus (HPV) infection [10,14,15]. 

Papillomaviruses (Papillomaviridae) constitute a diverse group of non-enveloped dsDNA viruses that exhibit tropism towards epithelial cells. More than 150 different types of papillomaviruses have been sequenced, 60 of which infect animals [16,17]. In total, over 200 types of HPV have been described [18,19]. Due to their oncogenic potential, HPV viruses are usually divided into low- and high-risk types. High-risk HPV viruses pose a much greater threat to human health than low-risk types [20,21]. The World Health Organization (WHO) identifies 12 types of HPV (16, 18, 31, 33, 35, 39, 45, 51, 52, 56, 58, 59) as a high risk for developing cancer, whereas HPV16 and HPV18 are considered the most oncogenic and their presence is detected in over 70% of cervical cancer cases. Besides cervical cancer, high-risk HPV viruses also cause other anogenital malignancies such as cancers of the anus, vulva, vagina, and penis [22,23]. HPVs are also believed to be responsible for “head and neck” cancers (pharynx, larynx, tongue) [23,24]. Low-risk HPV viruses (e.g., HPV6, 11, 42, 43, 44, 51, 53, 83) infecting mucosal epithelium usually do not cause malignancy, but rather benign proliferative changes such as skin warts, common warts, and flat warts [16,25,26]. Low-risk HPV types are rarely found in cancer. They are identified in malignant lesions indicating co-infection with high-risk HPV viruses, and, only in some cases, also cause lesions in the larynx and respiratory tract. For example, recurrent respiratory papillomatosis (RRP) has characteristic warts in the upper respiratory tract. The disease causes hoarseness, loss of voice, and impaired airway patency. It mainly develops in people with a weakened immune system and possibly with genetic predispositions.

The HPV genome encodes six early genes (E1, E2, E4, E5, E6, E7) which regulate viral transcription and genome replication and two late genes (L1, L2) which encode two capsid proteins [27]. The E6 oncoprotein from high-risk HPVs mediates the inactivation of tumor suppressor p53, which plays an essential role in preventing cancer development. In HPV-infected cells, the E7 oncoprotein interacts with pRb, which leads to premature entry into the S-phase of the cell cycle and uncontrolled cell division [28,29]. Upon HPV genome integration, E6 and E7 oncogenes often become deregulated, which leads to cell cycle deregulation, intensification of cell divisions, and carcinogenic progression [27]. Like other viruses, HPVs are intracellular parasites which utilize several hosts’ metabolic processes as well as the proteins coded by the viruses themselves in order to replicate. Therefore, effective antiviral therapeutics must interfere with virus-coded molecules but should not affect cellular processes. Targets for antiviral activity can be cell-interacting viral molecules, cellular receptors, viral nucleic acids, viral and cellular molecules, and virus-specific enzymes.

The therapeutic effect of *C. majus* latex against HPV is well known; however, the molecular mechanism behind this activity is still not fully understood. The latex of *C. majus* contains a variety of chemically active compounds such as proteins and alkaloids which can inhibit the replication of human papillomavirus by acting on different stages of its life cycle. The observed antiviral and antitumor properties of *C. majus* latex are often attributed to alkaloids contained therein; however, recent studies indicate that latex proteins may also play a significant role in its biological and pharmacological activities. In this study we have decided to use a novel approach where we divided latex components into two major groups: proteins and alkaloids, using the acetone protein precipitation technique. Thanks to this method we were able to point out more precisely than in the previous studies which group of compounds is involved in the observed phenomena. Henceforth, the crude *C. majus* latex will be referred to as S1 (Sample 1), protein fraction as S2 (Sample 2), and alkaloid-rich fraction (latex components free of proteins) as S3 (Sample 3). The aim of the study was to investigate the effect of the crude *C. majus* latex (S1) and the protein (S2) and alkaloid-rich (S3) fractions of the latex on HPV and on various stages of its replication cycle, including penetration into the cell, and transcription and translation of viral genes.

## 2. Results

### 2.1. The Verification of Latex Fraction Composition

Two latex fractions (protein and alkaloid-rich, S2 and S3, respectively) were obtained from crude *C. majus* latex using the acetone precipitation method. The content of the obtained fractions was analyzed using mass spectrometry. The results are presented in Table 1 and Table 2 and the Supplementary Materials. The obtained MS results for the alkaloid-rich fraction (S3) showed the presence of alkaloids, including coptisine, chelidonine, berberine, stylopine, protopine, and others (Table 1). They are mainly isoquinoline alkaloids, belonging to the following types of compounds: benzophenanthridine (chelidonine) and protoberberine (coptisine, dihydroberberine, berberine, stylopine). The full list of identified alkaloids together with their MS spectra are provided in Table S1 and Figures S1 and S2. 

In the protein fraction of *C. majus* latex (S2), the presence of proteins involved in general metabolism and defense proteins such as polyphenol oxidase, superoxide dismutase, major latex protein (MLP), peroxiredoxin, reticuline oxidase, heat shock protein 70 (Hsp), and peroxidase (POX), was detected (Table 2). 

### 2.2. Cytotoxicity Assay

It has been previously proven that the crude *C. majus* latex is highly cytotoxic; therefore, before proceeding with the research on antiviral activity involving cell cultures, the first step was to determine the cytotoxicity of the crude latex (S1), protein fraction (S2), and alkaloid-rich fraction (S3) against HeLa and HaCaT cells. Using a series of dilutions, the maximum non-toxic concentration (MNTC) of each sample was established. In the case of HeLa and HaCaT cells, the MNTC corresponded to a dilution of 10^−3^, where the protein concentration in the samples (S1, S2) was 0.8 µg/mL. These MNTCs of the samples were selected for further use in cell culture research. At this concentration of the examined samples, the treated cells exhibited a viability close to 100% compared to the untreated cells (control). Using samples at MNTCs in experiments involving cell cultures eliminated false positive or false negative results which may otherwise occur due to the use of cytotoxic concentrations of samples and would reduce the number of the tested cells. 

**Figure 1:**
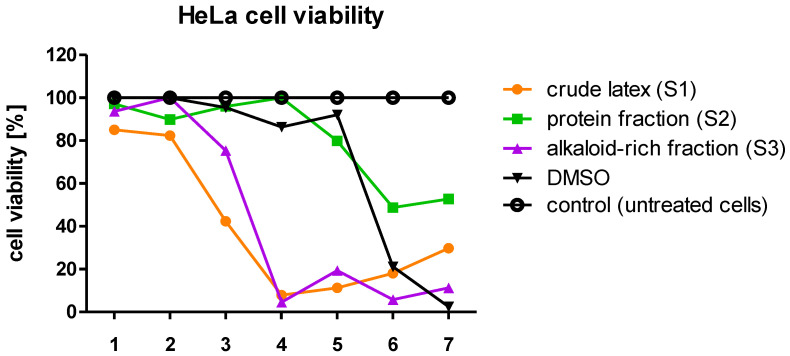
Effect of crude *C. majus* latex (S1), protein fraction (S2), and alkaloid-rich fraction (S3) on the viability of HeLa cells. Consecutive numbers (1–7) on the x axis correspond to different protein concentrations in the crude latex (S1) and protein fraction (S2) applied on cells: 1 = 4.0 × 10^−4^ mg/mL, 2 = 8.0 × 10^−4^ mg/mL, 3 = 1.6 × 10^−3^ mg/mL, 4 = 8.0 × 10^−3^ mg/mL, 5 = 1.6 × 10^−2^ mg/mL, 6 = 4.0 × 10^−2^ mg/mL, 7 = 8.0 × 10^−2^ mg/mL. In the case of alkaloid fraction (S3), consecutive numbers (1–7) correspond to its different dilutions: 1 = 5 × 10^−4^, 2 = 10^−3^, 3 = 2 × 10^−3^, 4 = 10^−2^, 5 = 2 × 10^−2^, 6 = 5 × 10^−2^, 7 = 10^−1^. They were obtained using series of dilutions from concentrated samples. Among the tested samples, the strongest cytotoxicity was shown by the crude latex and the alkaloid-rich fraction in the range of 8.0 × 10^−2^–8.0 × 10^−3^ mg/mL (numbers 4–7). The protein fraction showed the lowest toxicity towards HeLa cells in all analyzed concentrations. All tested samples in the concentration range of 8.0 × 10^−4^–4.0 × 10^−4^ mg/mL (numbers 1–2) exhibited the lowest cytotoxicity.

**Figure 2:**
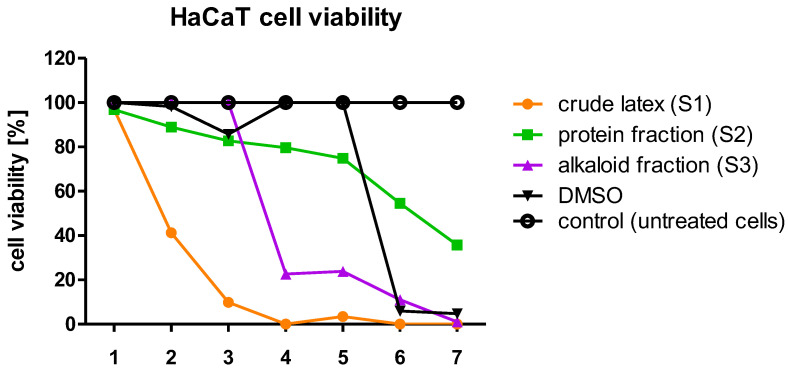
Effect of crude *C. majus* latex, protein fraction, and alkaloid-rich fraction on HaCaT cell viability. Consecutive numbers (1–7) on the x axis correspond to different protein concentrations in the crude latex (S1) and protein fraction (S2) applied on cells: 1 = 4.0 × 10^−4^ mg/mL, 2 = 8.0 × 10^−4^ mg/mL, 3 = 1.6 × 10^−3^ mg/mL, 4 = 8.0 × 10^−3^ mg/mL, 5 = 1.6 × 10^−2^ mg/mL, 6 = 4.0 × 10^−2^ mg/mL, 7 = 8.0 × 10^−2^ mg/mL. In the case of alkaloid fraction (S3), consecutive numbers (1–7) correspond to its different dilutions: 1 = 5 × 10^−4^, 2 = 10^−3^, 3 = 2 × 10^−3^, 4 = 10^−2^, 5 = 2 × 10^−2^, 6 = 5 × 10^−2^, 7 = 10^−1^. They were obtained from series of dilutions from concentrated samples. Among the tested samples, the crude latex and the alkaloid-rich fraction showed the strongest cytotoxicity in the range of 8.0 × 10^−3^–8.0 × 10^−2^ mg/mL (numbers 4–7), and the protein fraction exerted lowest toxicity against HaCaT cells in this concentration range. Most of the tested samples in the range of 4.0 × 10^−4^–1.6 × 10^−3^ mg/mL (numbers 1–3) showed the lowest cytotoxicity.

The results of the conducted experiments showed that the crude *C. majus* (S1) latex and the alkaloid-rich fraction (S3) were the most toxic for both HeLa and HaCaT cells (Figure 1 and Figure 2). HeLa and HaCaT cells treated with the highest concentration of the protein fraction (S2) had a viability of approx. 40–50% relative to untreated cells (control). In the range of 8.0 × 10^−3^–8.0 × 10^−2^ mg/mL, the crude latex and the alkaloid-rich fraction were highly toxic to HeLa and HaCaT cells. The viability of HeLa cells treated with such samples was approx. 10–20%, and HaCaT cells approx. 0–20%. Most samples in the range of 4.0 × 10^−4^–1.6 × 10^−3^ mg/mL showed the lowest cytotoxicity, and the treated cells had a viability of 80–100% for HeLa cells (Figure 1) and 40–100% for HaCaT cells (Figure 2).

### 2.3. PsV-HPV Capsid Protein Stability

**Figure 3:**
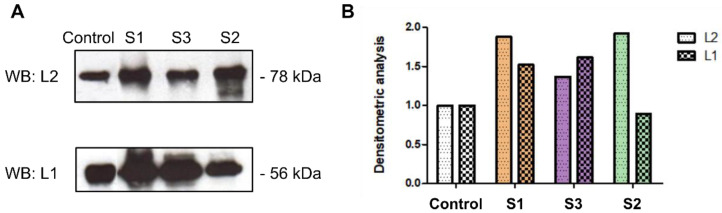
Effect of *C. majus* latex (S1), protein fraction (S2), and alkaloid-rich fraction (S3) on the stability of structural proteins L1 and L2 of the HPV capsid. HPV pseudovirions were treated with crude latex (S1) and its fractions (S2, S3), and incubated for 3 h. (**A**) Representative protein immunodetection result obtained by Western blot using specific α-L1 and α-L2 antibodies. S1—PsV-HPV particles treated with crude latex, S2—PsV-HPV particles treated with the protein fraction, S3—PsV-HPV particles treated with the alkaloid-rich fraction, Control—untreated PsV-HPV particles. After immunodetection, a similar amount of L1 and L2 proteins was observed for all tested samples, which proves that the crude *C. majus* latex, alkaloid-rich fraction, and protein fraction do not degrade PsV-HPV capsid proteins. (**B**) Results of densitometric measurements of signals obtained on the film after immunodetection.

HPV pseudovirions (PsV-HPVs) and permissive cell cultures (HaCaT) susceptible to HPV infection, served as a model for studying the HPV capsid protein stability, virus attachment, and entry in the presence of crude latex and its components (Figure S3). PsV-HPVs were treated with the concentrated crude *C. majus* latex (S1), protein fraction (S2), and alkaloid-rich fraction (S3) for 3 h to test if they affect the stability of the HPV capsid proteins (L1, L2). The stability of viral capsid structural proteins was determined using SDS-PAGE, followed by Western blotting. Immunodetection showed that the relative amounts of L1 and L2 proteins were similar in all tested samples and controls, which means that the crude *C. majus* latex and its fractions did not degrade capsid proteins of HPV pseudoviral particles (Figure 3).

### 2.4. Attachment of PsV-HPV Particles to Cell Surface

**Figure 4:**
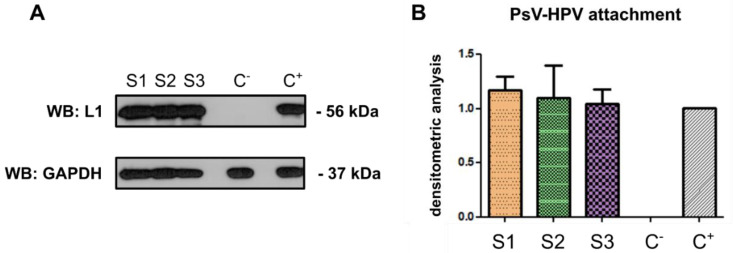
Effect of *C. majus* latex (S1), protein fraction (S2), and alkaloid-rich fraction (S3) on the attachment of HPV pseudovirions to the surface of HaCaT cells. HPV pseudoviral particles were applied to HaCaT cells that had previously been incubated with crude *C. majus* latex, alkaloid-rich fraction, or protein fraction. The amount of pseudoviral particles bound to the cells was then determined by Western blot and L1-specific antibody. (**A**) Representative Western blot result after immunodetection. S1—treatment with crude latex, S2—treatment with protein fraction, S3—treatment with alkaloid-rich fraction, C^−^—sample without PsV-HPVs, C^+^—untreated cells. (**A**) Clear signal for all tested samples indicates the lack of influence of the crude *C. majus* latex, alkaloid-rich fraction, and protein fraction on the stability of the PsV-HPV L1 capsid proteins. (**B**) Densitometric measurement of the signals obtained on the film after immunodetection, normalized to the signal for GAPDH.

Attachment of the virus to the cell surface is a prerequisite for initiation of an infection. We examined the effect of the three samples, the crude *C. majus* latex (S1), protein fraction (S2), and alkaloid-rich fraction (S3), on the attachment of HPV pseudovirions to the surface of HaCaT cells. The results are presented in Figure 4. The signal intensity for the L1 protein corresponds to the amount of HPV pseudoviral particles attached to cells. Post-immunodetection densitometric analysis showed no difference in the quantity of L1 proteins in samples treated with crude latex and test fractions versus the control (HaCaT cells with attached HPV particles, untreated with crude latex or its fractions) (Figure 4). These results show that neither the crude latex (S1) nor the protein fraction (S2) and alkaloid-rich fraction (S3) have any effect on the PsV-HPV attachment to the cell surface.

### 2.5. The Penetration of PsV-HPV Particles into HaCaT Cells

Due to the fact that neither the crude latex (S1) nor any of the tested *C. majus* latex fractions (S2, S3) degraded the PsV-HPV capsid proteins, in the next stage of the study we investigated whether the crude latex (S1) and its fractions (S2, S3), could affect the penetration of the HPV virus and impede the transport of the viral genome into the cell nucleus. The successful penetration marks the beginning of the virus life cycle inside the cell; hence, the measurement of penetration can be called virus infectivity [31]. 

**Figure 5:**
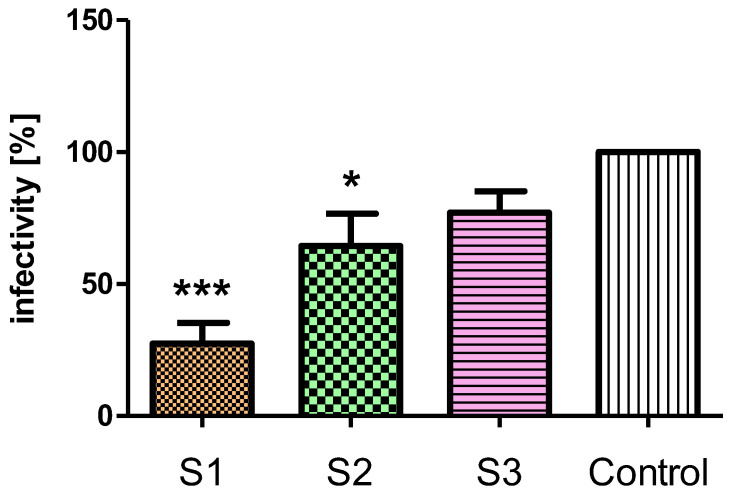
The influence of crude *C. majus* latex (S1), protein fraction (S2), and alkaloid-rich fraction (S3) on the infectivity of HPV pseudovirions in HaCaT cells. PsV-HPVs and non-toxic concentrations of crude latex, alkaloid-rich fraction, and protein fraction were added to the cells, and after 48 h of incubation the luminescence level was measured using a spectrophotometer. The luminescence intensity of a given sample corresponded to the infectivity of the virus in HaCaT cells. The lowest level of infectivity (the lowest intensity of luminescence) was observed for cells treated with the crude latex (27.5%). For the cells treated with alkaloid-rich fraction and protein fraction, the intensity of luminescence equaled 77.1% and 64.4%, respectively. S1—cells treated with crude latex, S2—cells treated with protein fraction, S3—cells treated with alkaloid-rich fraction, Control—untreated cells. * *p* < 0.05; *** *p* < 0.001.

The results indicated that the crude *C. majus* latex, alkaloid-rich fraction, and protein fraction reduce the infectivity of HPV pseudoviral particles in HaCaT cells. PsV-HPV penetration was reduced by 22.9% for the treatment of cells with the alkaloid-rich fraction, by 35.6% for the protein fraction, and the highest level of infection was decreased by the crude latex by 72.5% (Figure 5).

### 2.6. Expression of E6 and E7 Viral Genes in HeLa Cells at the mRNA Level

The next stage of the research was to verify whether the antiviral activity of the *C. majus* latex may be also caused by the influence on the expression of E6 and E7 oncogenes of the HPV virus at the mRNA level. The research was carried out on HeLa cells, derived from cervical cancer cells transformed with HPV18 virus, which express the viral E6 and E7 genes. Cells were incubated with non-toxic concentrations of crude latex (S1) and its fractions (S2, S3), and then RNA was isolated from them. After reverse transcription, cDNA was used as a template in real-time PCR with primers specific for HPV E6 and E7 oncogenes, and for the reference gene (GAPDH).

**Figure 6:**
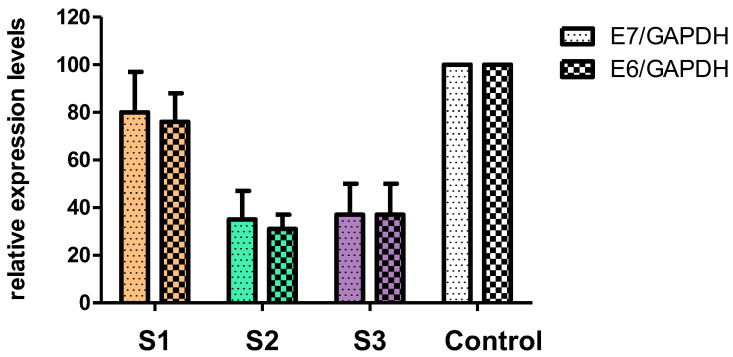
Effect of *C. majus* latex (S1), protein fraction (S2), and alkaloid-rich fraction (S3) on the expression of E6 and E7 viral oncogenes in HeLa cells. HeLa cells expressing E6 and E7 viral oncogenes were treated with the tested samples. RNA was isolated and transcribed into cDNA, which served as a template in real-time PCR using primers specific for the E6 and E7 genes and the GAPDH which served as reference gene. The lowest level of E6 and E7 transcripts was observed in cells treated with the protein fraction (E6 = 28%, E7 = 24%), was higher in cells treated with the alkaloid-rich fraction (E6 = 37%, E7 = 37%), and was the highest in cells treated with the crude *C. majus* latex (E6 = 76%, E7 = 80%) compared to untreated cells. S1—cells treated with crude latex, S2—cells treated with protein fraction, S3—cells treated with alkaloid-rich fraction, Control—untreated cells.

The results of the research showed that the mRNA level of E6 and E7 genes were the lowest in cells treated with the protein fraction of *C. majus* latex (S2) (reduction by 72% for E6 and by 76% for E7). When the alkaloid-rich fraction (S3) was administered to the cell culture, the mRNA level was reduced by 63% for E6 and E7, compared to the untreated cells (control). Crude latex (S1) showed the lowest decrease of 24% for E6 and 20% for E7 (Figure 6). Subsequently, we investigated how the crude latex (S1), protein fraction (S2), and alkaloid-rich fraction (S3) affected the expression of the viral E6 and E7 genes at the protein level.

### 2.7. Expression of E6 and E7 Viral Genes in HeLa Cells at the Protein Level

**Figure 7:**
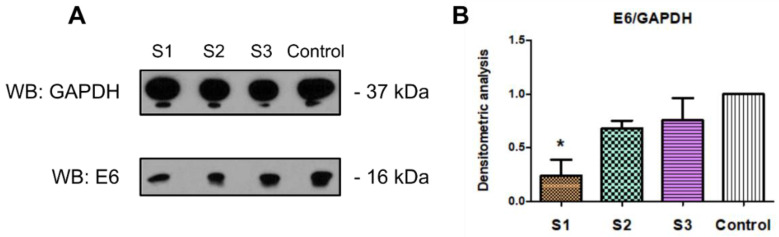
Influence of crude *C. majus* latex (S1), protein fraction (S2), and alkaloid-rich fraction (S3) on the expression of the viral E6 oncogene at the protein level in HeLa cells. Non-toxic concentrations of crude latex, alkaloid-rich fraction, and protein fraction were administered on HeLa cells. After 48 h of incubation, proteins were isolated, which were then separated by SDS-PAGE. Immunodetection of the E6 protein and the GAPDH as a reference protein was performed using the Western blot and specific antibodies. The densitometric measurement showed that the crude *C. majus* latex decreased the level of HPV E6 protein the most (reduction by 76.3%). Moreover, the protein fraction lowered the level of E6 protein by 32.1% and the alkaloid-rich fraction by 24.5%. S1—cells treated with crude latex, S2—cells treated with the protein fraction, S3—cells treated with the alkaloid-rich fraction, Control—untreated cells. (**A**) Representative Western blot of protein immunodetection. (**B**) Densitometric analysis of signals from protein fractions using ImageJ (NIH) software. * *p* < 0.05.

Densitometric measurements showed the lowest level of E6 protein in cells treated with crude latex (S1). The level of viral E6 protein was reduced by 76.3% compared to untreated cells (control). For the protein (S2) and alkaloid (S3) fractions, a lower level of E6 protein was also observed, reduced by 32.1% and 24.5%, respectively, compared to the control (Figure 7).

**Figure 8:**
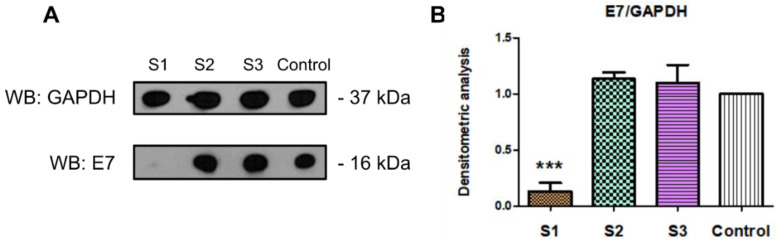
Effect of crude *C. majus* latex (S1), protein fraction (S2), and alkaloid-rich fraction (S3) on the expression of the viral E7 oncogene at the protein level in HeLa cells. Non-toxic concentrations of crude latex, alkaloid-rich fraction, and protein fraction were administered on HeLa cells. After 48 h of incubation, proteins were isolated, which were then separated by SDS-PAGE. Immunodetection of the E6 protein and the GAPDH as a reference protein was performed using the Western blot and specific antibodies. The densitometric measurement showed that only the crude *C. majus* latex reduced the level of HPV E7 proteins in HeLa cells (a reduction of 86.6%). The alkaloid-rich fraction and the protein fraction had no effect on the level of E7 protein synthesis. S1—cells treated with crude latex, S2—cells treated with the protein fraction, S3—cells treated with the alkaloid-rich fraction, Control—untreated cells. (**A**) Representative Western blot of protein immunodetection. (**B**) Densitometric analysis using ImageJ software (NIH). *** *p* < 0.001.

Similar to the E6 protein, the influence of the crude *C. majus* latex and its fractions on the level of the viral E7 protein was also investigated. Densitometric analysis showed the lowest level of E7 protein in cells treated with crude latex. E7 protein levels were 86.6% lower comparing to untreated cells (control). The tested latex fractions (alkaloid, protein) did not reduce the level of E7 protein synthesis (Figure 8).

Table 3 shows that the crude *C. majus* (S1) latex inhibited the expression of E6 and E7 genes at the protein level most strongly. The protein (S2) and alkaloid-rich (S3) fractions of *C. majus* latex reduced the expression of viral oncogenes at the mRNA level for E6 (by 72% and 63%, respectively) and for E7 (by 76% and 63%, respectively). At the protein level, the protein fraction (S2) inhibited E6 expression more strongly (32.1% decrease) than the alkaloid-rich fraction (S3) (24.5% decrease). 

### 2.8. In Silico Docking of Selected C. majus Latex Alkaloids to p53 Protein

**Figure 9:**
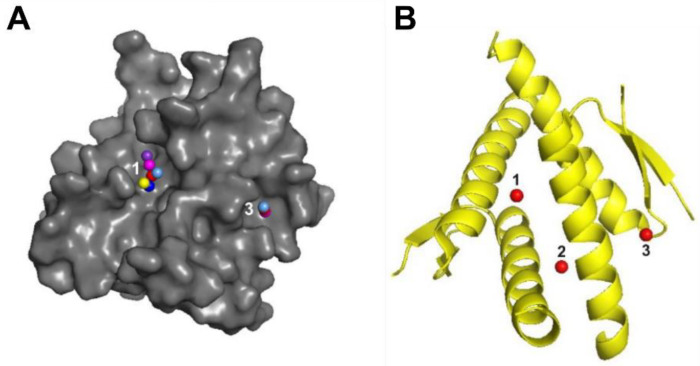
Identified p53 active sites to which selected alkaloids from *C. majus* latex were docked. (**A**) Colored dots represent the identified hot spots by different predictors. (**B**) p53 with all three active sites (1–3) to which berberine, dihydroberberine, and coptisine were docked. The illustrations were generated in PyMol software.

It has been proven that low-molecular-weight compounds can block the sites of interaction of the E6 protein with the p53 protein, which would result in its stabilization [32]. Therefore, molecular docking analysis was performed to verify whether selected *C. majus* latex alkaloids such as berberine, dihydroberberine, and coptisine could bind to the p53 protein (Table 4, Figure 9).

The docking of berberine, dihydroberberine, and coptisine showed a high similarity of the interaction at active sites 1 and 2 of the p53 protein. The binding energy in the three active sites was the lowest for coptisine. In silico analysis of the possibility of the binding of berberine, dihydroberberine, and coptisine molecules with the p53 protein showed that these molecules showed similar affinity for this protein (Table 5). This affinity is favored by the relatively rigid and flat structure of the molecules. The identified coordinates (x, y, z) of active sites used for the docking of ligands to p53 and amino acid residues found in these active sites are provided in Tables S3 and S4.

### 2.9. Nitric Oxide Secretion by RAW 264.7 Cells

**Figure 10:**
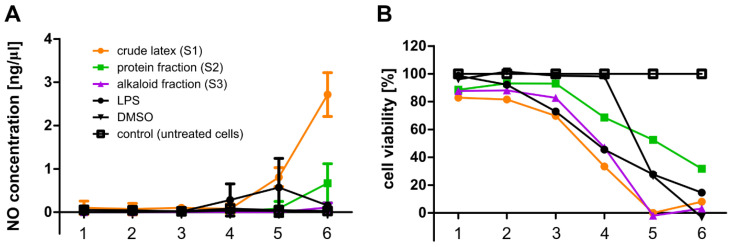
(**A**) Effect of crude *C. majus* latex, alkaloid-rich fraction, and protein fraction on nitric oxide (NO) secretion by RAW 264.7 cells. Consecutive numbers (1–6) on the x axis correspond to different protein concentrations in the crude latex (S1) and protein fraction (S2) applied on cells: 1 = 1.3 × 10^−5^ mg/mL, 2 = 6.4 × 10^−5^ mg/mL, 3 = 3.2 × 10^−4^ mg/mL, 4 = 1.6 × 10^−3^ mg/mL, 5 = 1.6 × 10^−2^ mg/mL, 6 = 8.0 × 10^−2^ mg/mL. In the case of alkaloid-rich fraction (S3), consecutive numbers (1–6) correspond to its different dilutions: 1 = 1.6 × 10^−6^, 2 = 8 × 10^−5^, 3 = 4 × 10^−4^, 4 = 2 × 10^−3^, 5 = 2 × 10^−2^, 6 = 10^−1^. They were obtained using series of dilutions from concentrated samples. Cells treated with the most concentrated samples of crude latex (S1) and the protein fraction (S2) released NO at a concentration of approximately 2.72 ng/μL and 0.67 ng/μL, respectively. These concentrations were highly cytotoxic and induced the death of most cells. No NO secretion was observed for cells treated with the samples at non-toxic concentrations. LPS-treated cells (100 ng/µL) (positive control) secreted NO at a level of 0.57 ng/μL. (**B**) Simultaneous analysis of the effect of crude *C. majus* latex (S1) and its fractions (S2, S3) on RAW 264.7 cell viability. Among the tested samples, the strongest cytotoxicity was shown for the crude latex and the alkaloid-rich fraction in the range 1.6 × 10^−3^–8.0 × 10^−2^ mg/mL (numbers 4–6). The protein fraction exhibited the lowest toxicity to RAW 264.7 cells in all analyzed dilutions compared to the rest of the samples. All tested samples in the range of 1.3 × 10^−5^–3.2 × 10^−4^ mg/mL (numbers 1–3) showed the lowest cytotoxicity.

In order to verify the influence of the crude *C. majus* latex (S1) and its fractions (S2, S3) on the induction of the inflammatory reaction, the secretion of nitric oxide (NO) by RAW 264.7 cells was investigated using the Griess reagent (Sigma-Aldrich, St. Louis, MO, USA). NO is one of the mediators of the inflammatory response. The results showed that under the influence of crude latex (S1) at two selected concentrations (1.6 × 10^−2^ mg/mL, 8.0 × 10^−2^ mg/mL), cells of the RAW 264.7 line released NO at a concentration of approximately 0.81 ng/μL and 2.72 ng/μL, respectively. The observed release of NO occurred only in the crude latex samples, which were highly toxic (1.6 × 10^−3^–8.0 × 10^−2^ mg/mL) and caused the death of most cells. No release of NO was observed in the presence of crude *C. majus* latex and the tested fractions at non-toxic concentrations for cells (Figure 10A).

At the same time, during the experiments using the Griess test, the cytotoxicity of the tested samples (S1, S2, S3) against RAW 264.7 cells was determined. The results of the cytotoxicity analyses showed that the crude *C. majus* latex (S1) and the alkaloid-rich fraction (S3) were both more toxic to RAW 264.7 cells at all tested concentrations than the protein fraction (Figure 10B). Results showed that crude latex (S1) and alkaloid-rich fraction (S3) in the range of 1.6 × 10^−3^–8.0 × 10^−2^ mg/mL were the most cytotoxic. The viability of RAW 264.7 cells was approximately 0–40%. All samples in the range of 1.3 × 10^−5^–3.2 × 10^−4^ mg/mL showed the lowest cytotoxicity and the treated cells had a viability of 70–100%. The cytotoxicity tests of the crude *C. majus* latex (S1) and its fractions (S2, S3) were also performed in the analysis of proinflammatory cytokines (TNFα, IL-6), and the obtained results were consistent with those described above, i.e., the crude latex (S1) and the alkaloid-rich fraction (S3) showed the strongest cytotoxicity (Figure 10B).

### 2.10. Secretion of Proinflammatory Cytokines by RAW 264.7 Cells

In order to test the effect of *C. majus* latex and its fractions on the induction of an inflammatory reaction, apart from the level of NO secretion, their influence on the secretion of two pro-inflammatory cytokines by RAW 264.7 cells was also examined. Tumor necrosis factor (TNFα) and interleukin 6 (IL-6) levels secreted by cells treated with crude latex and tested fractions were analyzed. Simultaneously, alongside the experiments analyzing the secretion of pro-inflammatory cytokines (TNFα, IL-6), the cytotoxicity of the tested samples (S1, S2, S3) against RAW 264.7 cells was examined.

**Figure 11:**
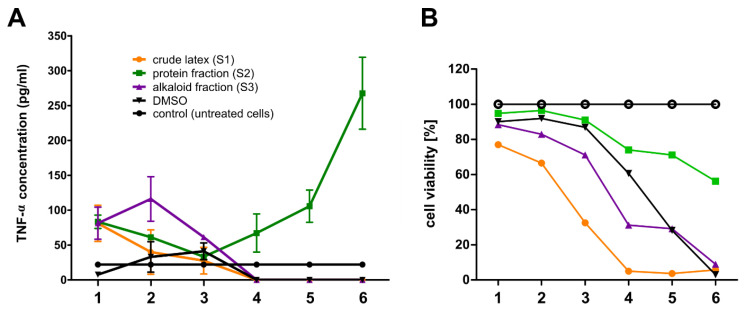
(**A**) Effect of crude *C. majus* latex (S1), protein fraction (S2), and alkaloid-rich fraction (S3) on tumor necrosis factor secretion by RAW 264.7 cells. Consecutive numbers (1–6) on the x axis correspond to different protein concentrations in the crude latex (S1) and protein fraction (S2) applied on cells: 1 = 1.6 × 10^−4^ mg/mL, 2 = 4.0 × 10^−4^ mg/mL, 3 = 1.6 × 10^−3^ mg/mL, 4 = 8.0 × 10^−3^ mg/mL, 5 = 1.6 × 10^−2^ mg/mL, 6 = 8.0 × 10^−2^ mg/mL. In the case of alkaloid-rich fraction (S3), consecutive numbers (1–6) correspond to its different dilutions: 1 = 2 × 10^−4^, 2 = 5 × 10^−4^, 3 = 2 × 10^−3^, 4 = 10^−2^, 5 = 2 × 10^−2^, 6 = 10^−1^. They were obtained using series of dilutions from concentrated samples. The protein fraction (S2) stimulated the secretion of TNFα by macrophages at the level of 267.7 pg/mL while maintaining approx. 60% cell viability. LPS-treated cells (100 ng/µL) (positive control) secreted TNFα at a level of 213.6 µg/mL. (**B**) Simultaneous analysis of the effect of crude *C. majus* latex, alkaloid-rich fraction, and protein fraction on RAW 264.7 cell viability. Among the tested samples, the strongest cytotoxicity was shown for the crude latex and the alkaloid-rich fraction in the range of 8.0 × 10^−3^–8.0 × 10^−2^ mg/mL (numbers 4–6). The protein fraction showed the lowest toxicity to RAW 264.7 cells in all analyzed concentrations.

The results showed that the protein fraction (S2) most strongly stimulated the secretion of tumor necrosis factor (TNFα) by macrophages at the level of 267.7 pg/mL while maintaining partial cell viability (approx. 60% viability) (Figure 11A). The crude latex (S1) and the alkaloid-rich fraction (S3) also stimulated TNFα secretion, but at a lower level due to their cytotoxicity to RAW 264.7 cells (Figure 11B).

**Figure 12:**
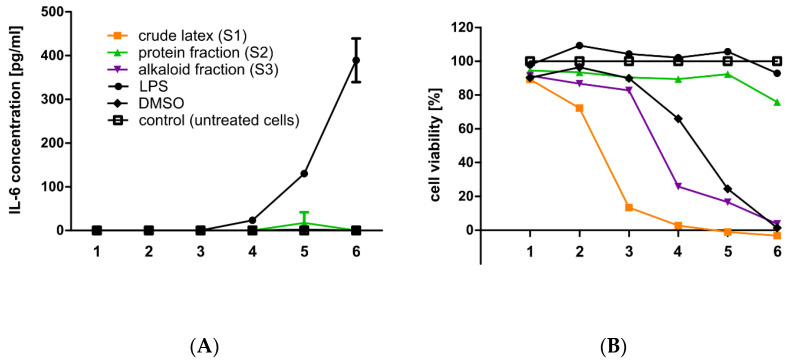
(**A**) Effect of crude *C. majus* latex (S1), protein fraction (S2), and alkaloid-rich fraction (S3) on the secretion of interleukin 6 (IL-6) by RAW 264.7 cells. Consecutive numbers (1–6) on the x axis correspond to different protein concentrations in the crude latex (S1) and protein fraction (S2) applied on cells: 1 = 1.6 × 10^−4^ mg/mL, 2 = 4.0 × 10^−4^ mg/mL, 3 = 1.6 × 10^−3^ mg/mL, 4 = 8.0 × 10^−3^ mg/mL, 5 = 1.6 × 10^−2^ mg/mL, 6 = 8.0 × 10^−2^ mg/mL. In the case of alkaloid-rich fraction (S3), consecutive numbers (1–6) correspond to its different dilutions: 1 = 2 × 10^−4^, 2 = 5 × 10^−4^, 3 = 2 × 10^−3^, 4 = 10^−2^, 5 = 2 × 10^−2^, 6 = 10^−1^. Cells treated with crude latex and its fractions in all dilution ranges tested (10^−1^–2 × 10^−4^) did not secrete IL-6 in detectable amounts. LPS-treated cells (100 ng/µL) (positive control) secreted IL-6 at a level of 424.7 pg/μL. (**B**) Simultaneous analysis of the effect of crude *C. majus* latex, alkaloid-rich fraction, and protein fraction on RAW 264.7 cell viability. Among the tested samples, the strongest cytotoxicity was shown for the crude latex and the alkaloid-rich fraction in the range of 8.0 × 10^−3^–8.0 × 10^−2^ mg/mL (numbers 4–6). The protein fraction showed the lowest toxicity to RAW 264.7 cells in all analyzed concentrations.

The second pro-inflammatory cytokine tested was interleukin 6 (IL-6). The results of the experiments showed no stimulation of RAW 264.7 cells to secrete IL-6 by the crude *C. majus* latex, nor by any of the analyzed fractions at all concentrations tested (Figure 12A).

## 3. Discussion

At the beginning of the study, it was assumed that the antiviral activity of *C. majus* latex against HPV may be related to the inhibition of at least one of several stages of the virus replication cycle. It was hypothesized that latex components can degrade viral particles before they enter the cell, block virion attachment to the cell surface, inhibit viral entry, and disrupt the viral replication cycle inside the cell at the stage of viral genetic material replication, transport to the cell nucleus, and transcription and translation of viral genes. It was also hypothesized that *C. majus* latex components can also affect the functioning of the immune system mediators. We tested the hypothesis point by point and we discuss the achieved results below. 

One of the important issues in the studying of potential antiviral compounds is the model to be used. The screening of antiviral compounds requires permissive cell-culture systems that allow penetration of the virus into the cell and optimal viral replication. It is difficult to find an in vitro model for investigating the HPV replication cycle, which would be tightly linked to differentiation of the epithelium. In our study, this limitation was partially overcome with the use of HPV pseudovirions (PsV-HPVs) and permissive cell cultures susceptible to infection. A single pseudovirion particle has a simplified structure in relation to the native virus occurring naturally in the environment. It consists of a viral capsid composed of two types of structural proteins (L1, L2); however, inside the capsid, instead of the viral genome, there is a pseudogenome, usually encoding a reporter gene. HPV pseudovirions are commonly used in the research of the structure of the viral capsid, virus entry, and assembly of progeny virions [19,33,34,35]. This research model allows the production of PsV-HPVs without the need for 3D raft cell cultures simulating multilayered epithelium, where the production time of pseudoviral particles is much more time-consuming [36,37].

The HPV pseudovirions produced in the HEK 293TT cell line were stable. The viral capsid proteins (L1, L2) separated in the PAA gel were not degraded, and the size of the pseudovirion particles in the transmission electron microscopy image was similar to the native HPV particles. Taking this into account, it was considered that the produced HPV pseudovirions are a suitable model for research on the effects of *C. majus* latex on the productive replication cycle of HPV.

In order to use HPV pseudovirions in experiments with the application of crude latex (S1) and its fractions (S2, S3), we investigated whether the tested samples degrade viral capsid proteins and, thus, could possibly destroy HPV pseudovirions. The literature data indicated that plant compounds can degrade viral proteins, influencing the shape, number, and size of viral particles [38,39].

After treatment of HPV pseudovirions with concentrated (undiluted) crude latex, no degradation of the viral capsid proteins L1 and L2 was observed. The viral proteins were also stable in the presence of the protein and alkaloid-rich fractions of the latex. The stability of the L1 and L2 proteins in the presence of latex and its fractions allows the viral particles to attach and enter the cell and transport their genetic material to the nucleus.

Since the concentrated *C. majus* latex (S1) and its fractions (S2, S3) had no effect on the stability of pseudovirions, it was concluded that the non-toxic concentrations used in further experiments would also have no effect on HPV pseudovirions. 

One of the goals of the research was to find out which group of compounds is responsible for specific biological properties of *C. majus* latex. They could be macromolecules such as proteins, low-molecular-weight compounds such as alkaloids, or maybe both groups of compounds working together in crude latex. Therefore, the crude latex (S1) was separated into protein (S2) and alkaloid (S3) fractions. Determination of the exact composition of the obtained fractions was important for understanding the molecular mechanism of antiviral activity of *C. majus* latex.

Numerous proteins have been identified in the protein fraction (S2). They are responsible for general metabolism and play a role in plant development, e.g., flower and fruit development. Some of the identified proteins belong to the pathogenesis-related (PR) protein family, members of which are involved in the plant’s response to various types of abiotic and biotic stressors. The MLP protein is a highly representative protein of *C. majus* latex and is present in the plant at various stages of its development up to fruit maturation [8,30]. This protein contains in its structure a conservative hydrophobic pocket that has the potential to bind low-molecular-weight compounds [40]. Results from our previous study showed that the hydrophobic pocket of MLP could bind with high affinity *C. majus* latex alkaloids such as berberine, 8-hydroxychelerythrine, and dihydroberberine [41]. This suggested a possible synergistic action of proteins and low-molecular-weight compounds contained in *C. majus* latex [8]. In addition, GRP proteins were detected in the protein fraction of *C. majus* latex, which are synthesized in the plant in response to stress and are associated with various post-transcriptional regulation processes [42,43,44]. Proteins identified in the protein fraction of *C. majus* latex are consistent with recently published reports [30,45]. The presence of defense proteins suggests that they may play an antiviral role in the latex. Plant PR proteins with antiviral activity against viruses such as HSV-2 and HIV have been previously described [39,46].

The second fraction isolated from the latex was the alkaloid-rich fraction (S3). The presence of the identified alkaloids in *C. majus*, as well as others, was previously described for the whole plant, but without focusing specifically on the latex components [30,47]. There are discrepancies in the literature on the alkaloid composition and concentrations in *C. majus*. This may be because the types of alkaloids and proteins as well as their concentrations change during the plant’s growth and development [45,47,48]. The synthesis of low-molecular-weight compounds, such as alkaloids, is closely dependent on the light and temperature; therefore, there are differences between the content of alkaloids and proteins from latex collected at different times of the day [15]. The results of the identification of *C. majus* latex alkaloids are consistent with the literature data [49]. The antiviral activity of alkaloids against HPV has been previously observed [50,51,52,53].

Latex from many plant species is highly toxic to cells. The cytotoxic effect of latex from *Euphorbia helioscopia*, *Euphorbia umbellata*, *Euphorbia antiquorum*, *Calotropis procera* and *Ficus carica* has been demonstrated towards cells of various cell lines [54,55,56,57,58]. *C. majus* latex was shown to be toxic towards hepatocytes [59,60]. *C. majus* crude latex is known to be cytotoxic to neoplastic cells [61,62]; however, there is no information in the literature regarding its toxicity towards HeLa cells. 

Analysis of the cytotoxicity of the tested samples (S1, S2, S3) towards the tested cell lines (HeLa, HaCaT, RAW 264.7) indicates that the crude latex (S1) showed the strongest cytotoxicity among all of them. This possibly suggests that there may be an interaction between proteins and low-molecular-components comprising the crude latex sample, where alkaloids are one of the most biologically active groups of compounds present in high amounts [15,63,64]. The toxicity of alkaloids towards cells is well known [65,66,67]. 

After determining the maximum non-toxic concentrations of the crude latex and its fractions, it was investigated how they affect selected stages of the viral replication cycle, starting with the viral particle attachment to the cell surface.

HPV attaches and enters cells through the HSPG (heparan sulfate proteoglycan) receptor and, most likely, through the α6 integrin as a secondary receptor [68]. When the interaction of the virus with the receptor is blocked, the entry is inhibited, and it is impossible for the virus to penetrate the cell [69,70,71]. Small-molecule compounds can interact with proteins and alter or block their activity; therefore, one of the mechanisms of antiviral activity of *C. majus* latex may be blocking the attachment of viral particles to the cell surface. This would prevent the virus from penetrating inside the cell and from going through the replication cycle properly. Plant latex components can act on cellular surface proteins that act as viral receptors to which the virus attaches before entering the cell. They can also act on proteins on the surface of viral capsids, preventing an interaction with cellular proteins. 

Using HaCaT cells, we investigated whether the *C. majus* latex components interfere with the entry of HPV pseudovirions into the cells. The results of the conducted experiments showed that none of the investigated samples (crude latex, alkaloid-rich fraction, protein fraction) inhibited the attachment of HPV pseudoviral particles to HaCaT cells. Therefore, the mechanism of the antiviral activity of *C. majus* latex against HPV is most likely not related to blocking the attachment of viral particles to the cell surface.

We investigated the effect of crude *C. majus* latex and its fractions (alkaloid, protein) on cell penetration and infectivity (ability to cause infection) of viral particles. The tested samples inhibit the infectivity of HPV pseudovirions in HaCaT cells. The results showed the most severely reduced infectivity of HPV pseudovirions in cells treated with crude latex. This lower level of infectivity compared to the control (untreated cells) may result from latex components blocking the viral particle entry into the cell and inhibiting the transport of viral genetic material into the cell nucleus. Plant compounds have been identified which interact with cell surface proteins and compete with viral particles, blocking their entry into cells. For example, chebulagic acid, a tannin obtained from *Terminalia chebula*, acts against HSV-1 by interacting with glycosaminoglycans on the cell surface, thus blocking the attachment of viral particles and the penetration into cells [72]. The strongest activity of crude latex may also result from the additive or synergistic effect of its components. The synergistic antiviral activity of plant extracts and latex components has been observed against different pathogens, including viruses such as influenza A virus [73,74]. Moreover, in our previous study we showed a synergistic inhibitory effect of *C. majus* latex alkaloids such as coptisine and berberine on cervical cancer cells (HeLa and C33A cell lines) [49]. 

A reduction in PsV-HPV infectivity may also result from the blockage of elements on the surface of the viral capsid through which the virus interacts with cell surface proteins. The compound 3HP-β-LG (3-hydroxyphthalic anhydride (3HP)-modified bovine beta-lactoglobulin) was shown to interact with a positively charged region of the HPV L1 protein, resulting in blocking virus entry into cells [75]. In addition, the L2N lipopeptide inhibits HPV entry into the cell by interacting with the N-terminus region of the L2 protein [76]. Additionally, many HIV entry inhibitors are known to act on the viral particle. For example, modified rutin inhibits HIV-1 infection at the cell entry stage, most likely by interacting with viral lipid envelope glycoproteins [77]. The GRFT (griffithsin) protein, isolated from algae, interacts with HIV-1 glycoproteins and blocks their attachment to cell surface proteins that act as viral receptors. This action results in blocking viral entry into cells [78,79].

The antiviral effect of the components of the tested samples may be effective at a later stage of virus entry into the cell and transport of its genetic material to the cell nucleus. The entry of HPV into the cell takes place through endocytosis. A compound called dynasore has been identified that blocks the entry of HPV by disrupting the activity of dynamin (GTPase). This results in the inability to develop endocytic vesicles [80].

The obtained results of the study showed that the infectivity of pseudoviral particles was most severely reduced in the presence of crude latex. It is known from the aforementioned experiments that HPV pseudovirions are not degraded after treatment with crude *C. majus* latex and its fractions (alkaloid, protein). In addition, PsV-HPVs can attach to cells treated with the test samples with no interference. Thus, *C. majus* latex components are likely to interact with PsV-HPV particles between cell entry and the pseudogenome reaching the cell nucleus.

The viral replication cycle in the cell can also be blocked by plant proteins. Antiviral properties against HIV have been demonstrated for proteins from various plant species such as *Chassalia parvifolia*, *Oldenlandia affinis*, *Phaseolus limensis*, *Phaseolus lunatus*, *Vigna sesquipedalis* cv. and *Phaseolus vulgaris* cv. [39,81,82,83,84]. The protein derived from *Stellaria media*, on the other hand, has antiviral properties against HSV [46]. The aforementioned proteins belong to PR (pathogenesis-related) or AMP (antimicrobial peptide) families, which are involved in the plant defense against pathogens. Crude *C. majus* latex contains similar proteins belonging to the family of PR proteins that can reduce the infectivity of viral particles [8].

The antiviral effect of the *C. majus* latex components may also be a result of blocking the expression of viral genes. The results of the analysis of the expression of viral genes at the mRNA level showed that the protein fraction (S2) showed the strongest activity, reducing the level of E6 transcripts by 72% and E7 by 76% (Table 3). This may be a result of the presence of proteins that can interact with RNA molecules, leading to their degradation. It is known that *C. majus* latex contains, among others, proteins with RNA-binding motifs [8,85]. One such protein is a glycine-rich protein (GRP). Its structure contains an RNA-recognition motif (RRM) through which it can interact with RNA. Moreover, in the crude *C. majus* latex and in the protein fraction, pathogenesis-related (PR) proteins, major latex proteins (MLPs), and other proteins are present, which possess properties that allow the binding and degrading of nucleic acids. The MLP protein belongs to the Bet v1 superfamily, which also includes PR-10 proteins, and the literature data indicate that the function of MLP proteins is similar to that of PR-10 proteins [40]. PR-10 proteins show ribonucleolytic properties and they are involved in a plant’s defense against viral pathogens. For example, a PR-10 protein from *Capsicum annuum,* called CaPR10, degraded viral RNA of *tobacco mosaic virus* (TMV). PR-10 genes were upregulated in plants infected with *cucumber mosaic virus* (CMV), TMV, and *tobacco etch virus* (TEV) [86,87,88]. They have also been shown to have antiviral properties by inhibiting virus entry into the cell and/or its replication [88]. The role of the MLP protein is probably similar to that of PR-10 proteins and may involve participation in the antiviral activity of latex [8]. By binding to mRNA molecules, these proteins can block their translation, transport them to the cytoplasm, and lead to their degradation, disrupting the viral replication cycle [44]. Proteins which interact with DNA can bind to the promoters of viral genes and block their transcription. Therefore, the mechanism responsible for lowering mRNA and protein levels may also degrade viral DNA or block the translation of viral mRNA. Thus, the GRP, MLP, and other proteins contained in the protein fraction could bind and degrade the E6 and E7 transcripts, which resulted in a reduction in their amount relative to the control (untreated cells). For crude latex (S1), the reduction in transcripts observed (E6 = 24% reduction, E7 = 20% reduction) was lower than that of the protein fraction (E6 = 72% reduction, E7 = 76% reduction). The activity of the proteins contained in *C. majus* latex and their ability to bind RNA may depend on the presence of alkaloids that can interact with protein active sites. Based on bioinformatic analysis (docking of alkaloids to proteins) it was previously shown that alkaloids such as dihydroberberine, berberine, and chelerythrine exhibit strong affinity for the active site of the major latex protein (MLP) [41]. The level of E6 and E7 transcripts was also strongly decreased by the alkaloid-rich fraction. The mRNA level for both E6 and E7 oncogenes was reduced on average by 63% compared to the control (untreated cells). It is possible that alkaloids, by interacting with, for example, cellular transcription factors or enzymes responsible for transcription, can reduce the level of viral mRNA. Berberine has been shown to block the transcription factor AP-1 (activator protein 1), which is responsible for the expression of E6 and E7 viral oncogenes. As a result, the expression of these genes is inhibited and, consequently, the level of viral proteins is lowered [51]. A similar mechanism of action may be responsible for lowering the levels of E6 and E7 transcripts treated with the *C. majus* alkaloid-rich fraction, which contains berberine. The alkaloids contained in *C. majus* crude latex and the alkaloid-rich fraction may also affect the transcription and translation of viral genes. Berberine was observed to partially inhibit the transcription and translation of HSV genes [89]. Moreover, it interferes with the replication of influenza A virus. The mechanism is based on blocking the export of viral ribonucleoprotein from the cell nucleus by inhibiting the signaling pathway with the participation of MAPK/ERK kinases [90].

The crude *C. majus* latex (S1) and the tested latex fractions (S2, S3) also influenced the expression of viral genes at the protein level. Western blot immunodetection and densitometric analyses showed that the crude latex (S1) had the strongest effect and decreased viral proteins by 76.3% for E6 and 86.6% for E7. A 32.1% decrease in the level of E6 protein was also observed in cells treated with the *C. majus* protein fraction and a 24.5% in cells treated with the alkaloid-rich fraction (Figure 7 and Figure 8). No effect of the tested *C. majus* latex fractions on the level of E7 protein was observed. This may be due to the different stability of both proteins and their post-translational modifications. The E7 protein is phosphorylated differently; unlike the E6 protein, it can perform its complex functions during the cell cycle [91]. Such additional phosphorylation of the protein may also increase its stability and durability [92]. 

There are many other factors that can influence the differences between mRNA and protein levels. Discrepancies may be a result of post-transcriptional modifications responsible for mRNA processing. Transcription and translation efficiency as well as mRNA and protein stability (in vivo half-lives) are also important in this matter [93,94].

The obtained results suggest a disturbance in the process of transcription and translation of viral genes in the presence of crude *C. majus* latex as well as its components. It is worth noting that the study analyzed two selected viral genes in terms of the influence of crude latex and the fractions tested on the mRNA and proteins encoded by them. The effect of crude *C. majus* latex on the remaining HPV genes is unknown, but it may be assumed that it is not selective for viral genes and the entire process of transcription and translation in cells may be disrupted.

One hypothesis on the mechanism behind the antiviral and antitumor activity of *C. majus* latex may be the blocking of the interaction of the HPV E6 protein with the cellular p53 protein by latex components. In order to lay the foundation to test this hypothesis, in silico molecular docking of selected alkaloids (ligands) to p53 was performed. The results showed that *C. majus* latex alkaloids, such as berberine, dihydroberberine, and coptisine, exhibit strong affinity for the active site of p53 (Table 5, Figure 9). A great similarity was observed in the strength of the interaction with the studied protein for the three alkaloids tested. It has been shown that small-molecule compounds can interact with the active site of the p53 protein, preventing binding to the viral E6 protein [32,95]. The E6AP complex binding site on the surface of p53 is then blocked by the attached low-molecular-weight compound. Hence, p53, a cell cycle regulator, is not degraded by the viral E6 protein, which in turn leads to inhibition of the proliferation of virus-infected cells and the induction of apoptosis. A similar mechanism of action might be responsible for the antiviral activity of *C. majus* latex and alkaloids contained therein; however, in vitro studies are required to confirm these findings. 

None of the tested samples completely blocked the infection of cells with HPV pseudoviral particles. Therefore, we investigated how the crude *C. majus* latex and its alkaloid and protein fractions act on the cells of the immune system. One hypothesis was that raw latex components do not act directly on the virus, but indirectly induce an inflammatory response, modifying the host’s immune system.

In order to verify whether the raw latex and the tested fractions can stimulate the inflammatory response, a system based on macrophage-like cells of the RAW 264.7 cell line was employed, which is often used in similar analyses [64,96,97]. Like human macrophages, RAW 264.7 cells can secrete inflammatory mediators during inflammation, which were considered markers of this process. Our attention was focused on three selected mediators of the inflammatory reaction: nitric oxide (NO), tumor necrosis factor alpha (TNFα), and interleukin 6 (IL-6) [96,98]. Simultaneously with the measurements of the concentration of inflammatory mediators secreted by the cells, the cytotoxicity of diluted samples of raw latex and its protein and alkaloid-rich fractions on RAW 264.7 cells was analyzed. The results of the experiments showed that in the case of NO measurement, only cells treated with the lowest dilutions (the most concentrated samples) of the crude latex released an increased amount of NO compared to untreated cells (control). It is worth noting that such concentrations of crude latex (e.g., 1.6 × 10^−2^ mg/mL; 8.0 × 10^−2^ mg/mL), were toxic to RAW 264.7 cells, which did not survive the 48 h incubation period. The induction of NO secretion at a concentration of 0.81 ng/μL by cells treated with crude *C. majus* latex at a concentration of 1.6 × 10^−2^ mg/mL and 2.72 ng/μL in the case of a crude latex at a dilution of 8.0 × 10^−2^ mg/mL is likely the result of the cytotoxicity of these latex dilutions (Figure 10). In a cytotoxic environment, macrophages emit NO, which is a defensive reaction to unfavorable conditions. The production of NO by macrophages is toxic to these cells [99].

In order to measure the concentration of the investigated pro-inflammatory cytokines TNFα and IL-6, tests were performed using the enzyme-linked immunosorbent assay (ELISA) method on RAW 264.7 cells. TNFα and IL-6 are among the major cytokines involved in the inflammatory response [100]. TNFα is mainly produced by macrophages and is involved in the acute phase of the inflammatory response. It is responsible for the involvement of neutrophils and the activation of arachidonic acid metabolism [101]. Among the two analyzed pro-inflammatory cytokines, no increase in the secretion of interleukin 6 (IL-6) by RAW 264.7 cells after treatment with different concentrations of the tested samples (S1, S2, S3) compared to untreated cells (control) was observed. In the case of TNFα, an induction of the secretion of this factor at a concentration of 267.7 pg/mL by RAW 264.7 cells was observed when the cells were treated with the *C. majus* protein fraction at a concentration of 8.0 × 10^−2^ mg/mL. It should be emphasized that the protein fraction in this concentration was partially toxic to cells, and the cell viability was approx. 60%. In addition, the crude *C. majus* latex and the alkaloid-rich fraction also stimulated the secretion of TNFα, but at a lower level than the protein fraction. This was probably due to their cytotoxicity to RAW 264.7 cells, as macrophages secrete TNFα in response to cytotoxic environmental conditions [102]. Increased secretion of TNFα while maintaining partial cell viability may indicate pro-inflammatory properties of latex proteins. Among the three mediators of the inflammatory reaction, only the secretion of TNFα by cells treated with the protein fraction was increased compared to untreated cells (control). This may suggest an occurrence of an inflammatory reaction at the site of the crude latex application; however, further studies are necessary. The *C. majus* latex protein fraction, which increased the secretion of TNFα, contained a mixture of proteins; thus, studying the effect of separated proteins on the secretion of inflammatory mediators is a promising research path. The observed increased secretion of TNFα may be due to the presence of pro-inflammatory proteins in *C. majus* latex. Moreover, the latex of many plant species is known to contain allergenic substances which may induce cytokine secretion [103]. In *C. majus* latex, we have identified proteins with potential allergenic activity, such as polyphenol oxidase, which in eggplant acts as an allergen, and calmodulin-like proteins. Additionally, MLPs, which are highly representative proteins of *C. majus* latex, belong to the Bet v 1 superfamily of protein allergens (Table S2) [104,105,106]. 

To conclude, we did not observe the degradation of viral capsid proteins (L1, L2) after incubation of HPV pseudovirions with the crude *C. majus* latex (S1) and its fractions (S2, S3); hence, there is no effect of the tested samples on the stability of HPV structural proteins. Latex components partially inhibit the penetration of PsV-HPV particles into cells. Results obtained from cells treated with latex alkaloids (e.g., berberine and its derivatives) and proteins correlate with the lower levels of E6 and E7 viral oncogenes and oncoproteins. The protein fraction (S2) acted most strongly on E6 and E7 gene expression at the mRNA level. The crude latex and the alkaloid-rich fraction have strong cytotoxic properties against HeLa, HaCaT, and RAW 264.7 cells. The protein fraction of latex may induce an inflammatory response that stimulates the immune system and indirectly helps to combat viral infection; however, further studies are required. 

The obtained research results show for the first time the influence of particular groups of *C. majus* latex compounds on different stages of the HPV replication cycle and contribute to a better understanding of the molecular mechanism of action of *C. majus* latex on HPV. Presented findings indicate that both *C. majus* latex proteins and alkaloids play a role in latex antiviral properties. They pave the way for future investigations of the interactions between latex alkaloids and proteins in light of their antiviral activity.

## 4. Materials and Methods

### 4.1. Collection and Preparation of Plant Material

The *Chelidonium majus* L. latex (milky sap) was collected from the shoots of the plant during its flowering stage. The time of harvesting was around midday, during sunny days from May to June from several selected natural sites near the University Campus Morasko in Poznań. The sites of harvest were in close proximity to each other. The exact locations of the sample collection were as follows: location 1—52°27′53.7″ N, 16°55′53.5″ E; location 2—52°27′44.6″ N, 16°55′56.6″ E; location 3—52°27′36.8″ N, 16°56′32.9″ E. Latex was harvested from plants about 50 cm high, which were at a similar stage of development. The shoots of the plant were incised with a scalpel and the flowing yellow-orange juice was collected with an automatic pipette and resuspended in a buffer containing 10% glycerol and 0.1 M of Tris-HCl, pH 8.0 (buffer to sap ratio was 1:1). The samples were then placed in a Dewar box filled with liquid nitrogen for immediate freezing. The samples were stored at −80 °C for further use. Samples prepared in this manner were considered “crude” latex.

### 4.2. C. majus Crude Latex Fraction Separation

Each sample containing the harvested crude *C. majus* latex was centrifuged for 20 min at 4 °C at 14,000 rpm (25,000× *g*) to centrifuge the coagulation debris. The remaining supernatant was referred to as the crude *C. majus* latex sample (S1).

#### 4.2.1. Protein Fraction

The crude latex sample (S1), in order to precipitate the proteins, was treated with acetone frozen to the temperature of −20 °C in a volume ratio of 1:4 (e.g., 800 μL of acetone was administered to 200 μL of the S1), vigorously mixed, and incubated overnight at −20 °C. Then, the samples were centrifuged for 15 min at 14,000 rpm (25,000× *g*) at 4 °C. The supernatant was transferred to a new tube to remove acetone in a vacuum concentrator and obtain an alkaloid-rich fraction. The protein pellet remaining after centrifugation was also placed in the RVC 2-18 CDplus vacuum concentrator (Christ) to centrifuge the residual acetone. The dried pellet was resuspended in PBS with vigorous shaking. The sample prepared in this way (pellet suspended in PBS buffer) was treated as the protein fraction of *C. majus* latex (S2). Protein concentration was measured by the Bradford method. As a result, equal concentrations of proteins and alkaloids in both of the isolated fractions and in the crude latex were always used. The protein concentration in the obtained protein fraction was 0.8 μg/μL.

#### 4.2.2. Alkaloid-Rich Fraction (Latex Components Free of Proteins)

The supernatant obtained after the precipitation and centrifugation of proteins from the crude *C. majus* latex, containing mainly alkaloids and low-molecular-weight compounds, was transferred to a new tube. Then, in order to dispose of the acetone used for protein precipitation, it was evaporated in a RVC 2-18 CDplus vacuum concentrator (Christ) for 2 h at 20 °C. After removal of acetone, the PBS buffer was added to the remaining supernatant so that the final volume corresponded to the original volume of the crude latex from which the proteins were precipitated. The sample prepared in this way was a low-molecular-weight *C. majus* latex fraction devoid of proteins, which, for the sake of simplicity, was treated as an alkaloid-rich fraction (S3). As a result, the dilutions of crude latex and the alkaloid-rich fraction used did not differ in the concentration of alkaloids contained in them.

### 4.3. Protein Concentration Measurement

Protein concentration was determined by the Bradford method using a Bradford reagent (Bradford Reagent, Sigma-Aldrich) according to the manufacturer’s instructions (Bradford 1976). Measurements were made in 96-well plates using a Synergy™ H1 spectrophotometer (BioTek, Winooski, VT, USA). To the samples containing 5 µL of protein solution, 250 µL of Bradford’s reagent was added. The whole mixture was incubated for about 20 min at room temperature and the absorbance was measured at 595 nm. Wells containing only Bradford’s reagent were treated as blank. Protein concentration in the samples was determined on the basis of a standard curve made of bovine serum albumin (BSA) solutions.

### 4.4. SDS-PAGE and Protein Immunodetection Using Western Blot

Proteins and protein extracts (*C. majus* latex proteins, L1 and L2 viral proteins, etc.) were analyzed by electrophoretic separation in polyacrylamide gel under denaturing conditions (SDS-PAGE, sodium dodecyl sulfate polyacrylamide gel electrophoresis). For this purpose, a two-stage polyacrylamide gel was prepared. The top gel (stacking) with a concentration of 5% is used to concentrate the tested samples, and the proper separation of the samples takes place in the bottom (resolving) gel with an acrylamide concentration of 10–12%, depending on the molecular weight of the proteins tested [107]. Electrophoresis was performed at 40 mA and 130 V for 2 h in Laemmli buffer (0.125 M Tris, 0.96 M glycine, 0.5% SDS). After SDS-PAGE, the proteins in the gel were stained overnight in Coomassie brilliant blue at RT or transferred to a PVDF membrane (Amersham™ Hybond^®^ P Western blotting membranes, PVDF; GE Healthcare, Chicago, IL, USA) using a semi-dry transfer. The transfer occurred at an intensity dependent on the PVDF membrane area (0.8–1 mA/cm^2^ of membrane) from the cathode to the anode. The duration of the transfer depended on the molecular weight of the proteins transferred and was usually 70–90 min. After the transfer, the PVDF membrane was blocked by placing it in a solution of 5% skimmed milk powder (AppliChem) dissolved in 1× PBS-T buffer for 1 h at RT. The membrane was then immersed in the primary antibody solution and incubated overnight at 4 °C. The unbound antibody was washed by placing the PVDF membrane three times in PBS-T solution for 15 min at RT. Then, the membrane was incubated with an appropriate secondary antibody labeled with horseradish peroxidase for 1 h at RT. After incubation, the membrane was washed three times for 15 min in PBS-T. The ECL Prime Western Blotting System (GE Healthcare) reagent (GE Healthcare), according to the manufacturer’s instructions, and Medix XBU X-ray films (FOMA) were used for protein immunodetection. The time of exposure of the plates was determined experimentally (3 s–20 min). Primary antibodies used in the study: HPV18 E6 (G7): sc-365089; HPV18 E7 (F-7): sc-365035; GAPDH (6C5): sc-32233; HPV16 L1 (CAMVIR-1): sc-47699; HPV16 L2 (2JGmab#2): sc-65707. All primary antibodies were purchased from Santa Cruz Biotechnology. Secondary antibody: Chicken Anti-Mouse IgG H&L (HRP): ab6814 (Abcam, Cambridge, UK).

### 4.5. Densitometric Analysis

Changes in gene expression (E6, E7) at the protein level were densitometrically analyzed on the basis of the intensity of film blackening induced by a reaction catalyzed by the horseradish peroxidase conjugated with a secondary antibody. For this purpose, computer analysis with the use of ImageJ (NIH) software was used. The obtained numerical values were normalized to the values obtained for the reference protein GAPDH (loading control).

### 4.6. Identification of Proteins and Alkaloids Using Mass Spectrometry

Identification of proteins contained in crude *C. majus* latex (S1) and in the protein fraction (S2) was performed using mass spectrometry methods. 

Proteins were identified using tandem mass spectrometry (LC-ESI-MS/MS) in the Laboratory of Mass Spectrometry, Institute of Biochemistry and Biophysics, PAS, Warsaw, Poland. Stained protein bands were analyzed by liquid chromatography coupled to the LTQ Orbitrap XL (Thermo Fisher Scientific, Waltham, MA, USA). Excised gel fragments were placed in 1.5 mL Eppendorf tubes filled with 10% methanol and 2% acetic acid. The proteins were digested using trypsin. The generated peptides were concentrated, desalted on an RP-C18 precolumn (LC Packings, Coventry, UK), and further separated by the UltiMate nano-HPLC (LC Packings, San Francisco, CA, USA). The column outlet was directly coupled to a Nanospray ion source operating in a data-dependent MS to MS/MS switch mode. Identification of proteins matching with the *C. majus* CDS database (Cmajus 20150107_1; 209,790 sequences; 74,516,318 residues), obtained through the Department of Molecular Virology, Adam Mickiewicz University [30], was performed by using the Mascot database search engine (Matrix Science, London, UK; www.matrixscience.com (accessed on 24 January 2018)) with the following parameters: mass measurement error tolerance of 0.8 Da, 1 site allowed in the peptide that is not enzymatically cleaved, carbamidomethylation of cysteine residues, and possible oxidation of methionine. The obtained results, in the form of a list of proteins with the highest score and sequence coverage, also contained data on the molecular weights of the identified proteins and their isoelectric point (pI). 

Alkaloid-rich fraction analysis was performed at the Center for Advanced Technology, AMU using HPLC-MS. Alkaloids were identified by dr. Grażyna Bartkowiak from the Faculty of Chemistry, AMU. Full range mass spectra were recorded using the amaZon SL ion trap (Bruker Daltonik GmbH, Bremen, Germany) with an electrospray ion source in infusion mode. The sample was introduced into the ionization source at a flow rate of 3 µL min^−1^ using a syringe pump. The capillary voltage was −4.5 kV and the end plate offset was −500 V. The temperature of the source was 80 °C and the desolvation temperature was 250 °C. Nitrogen was used as the nebulizing and desolvation gas at a flow rate of 5 L/min and a drying temperature of 200 °C, and as the auxiliary gas helium. All mass spectra were measured in the positive ionization mode. The trapControl software and the Bruker Compass DataAnalysis 4.2 application were used for the processing and analysis of the spectra.

### 4.7. Cell Lines

Four types of cell lines were used in the research: HeLa, HaCaT, RAW 264.7, and HEK 293TT. Cells were grown in an incubator at 37 °C, 95% humidity, and 5% CO_2_ concentration. All cells of the cell lines used were grown in DMEM (Dulbecco’s Modified Eagle Medium), which is high in glucose, pyruvate, and glutamine (GlutaMAX™), and supplemented with 10% fetal bovine serum (FBS). Antibiotics: streptomycin (100 μg/mL) and penicillin (100 U/mL) were also added to the medium. Cells were passaged every 3–4 days to maintain culture continuity. 

### 4.8. Cytotoxicity Assay

The cytotoxicity of the tested *C. majus* latex fractions against the cells of the used cell lines was tested in 96-well plates using the PreMix WST-1 Cell Proliferation Assay System (TaKaRa, San Jose, CA, USA). The method is based on the conversion of bright-red tetrazole salts to dark-red formazan by mitochondrial dehydrogenase. This reaction only occurs in living cells in which this enzyme is active. In order to test the cytotoxicity of the crude *C. majus* latex and the tested fractions (alkaloid, protein), cells were seeded in a 96-well plate. Depending on the type of cell line used, different numbers of cells were seeded into a single well of the plate (HeLa: 3000 cells per well, HaCaT: 5000 cells per well, RAW 264.7: 5000 cells per well). Appropriately seeded cells were incubated overnight at 37 °C and 5% CO_2_ concentration. The medium was then removed and the crude latex and test fractions diluted in DMEM (100 µL/well) were added to the appropriate wells. Wells with cells in which only the DMEM was replaced with fresh medium were used as a negative control. The positive control consisted of wells with cells treated with DMSO, which at room temperature was cytotoxic. Cells were incubated with the diluted fractions for 48 h at 37 °C and 5% CO_2_. Then, 10 μL of PreMix WST-1 reagent was applied to all wells with tested samples, and then incubated for 1 h at 37 °C with 5% CO_2_. The “blank” samples were wells with no cells, but only DMEM and PreMix WST-1. Two-wavelength absorbance measurements were then performed (450 nm—actual measurement; 620 nm—reference wavelength) using a Synergy™ H1 reader (BioTek).

### 4.9. Cell Lysis and Protein Isolation

The culture vessels with the cells were transferred from an incubator (37 °C, 5% CO_2_) to ice, then the DMEM was removed, and the cells were washed with chilled PBS solution. The PBS solution was then removed and the chilled TAP cell lysis buffer (50 mM Tris-HCl, pH 7.5; 150 mM NaCl; 1 mM EDTA, pH 8.0; 10% glycerol; 1% NP-40) with added protease inhibitors was added to the cell wells. The culture dishes with the cells were incubated on ice for 10 min. The cells were then detached from the bottom of the wells of the culture vessel using sterile scrapers and transferred to chilled 1.5 mL Eppendorf tubes. Tubes with cells were incubated on ice for 15 min, with vigorous mixing every 5 min. Protein extracts prepared in this way were centrifuged for 20 min at 4 °C and 14,000 rpm (25,000× *g*). After this time, the obtained supernatant was transferred to sterile tubes, frozen, and stored at −20 °C until later use.

### 4.10. Griess Assay—Measurement of Nitric Oxide Concentration

The Griess colorimetric method was used to measure the concentration of nitric oxide (NO) secreted by RAW 264.7 cells treated with crude *C. majus* latex and the tested fractions. The Griess reagent (Sigma-Aldrich) is a solution of sulfanilic acid and α-naphthylamine in acetic acid, which changes the color of the solution to pink or red when there is a reaction with nitrites (NO^2−^).

To test the concentration of NO secreted by RAW 264.7 cells treated with crude *C. majus* latex (S1) and the tested fractions (S2, S3), cells were seeded in a 96-well plate at 5000 cells per well. Appropriately seeded cells were incubated overnight at 37 °C and 5% CO_2_ concentration. The medium was then removed and the crude latex diluted in DMEM and the test fractions (100 μL/well) were administered. Wells with cells in which only the DMEM was replaced with fresh medium were used as a negative control. The positive control consisted of wells with cells treated with lipopolysaccharide (LPS) at a concentration of 100 ng/μL, which induces an inflammatory response and stimulates NO secretion by macrophages. Cells were incubated with various concentrations of the tested samples (S1, S2, S3) for 48 h at 37 °C and 5% CO_2_ concentration. Then, 50 μL of medium from all test wells was transferred to a new 96-well plate. The “blank” samples were wells containing medium transferred from wells containing no cells. The empty wells were filled with NaNO_2_ standard in volumes of 10, 20, and 40 µL in duplicates, which were then filled up to 50 µL with DMEM. Subsequently, the Griess reagent was administered in the ratio of 1:1 (*v/v*) to all wells with the tested samples and to the “blank” samples. The prepared plate was protected from light and incubated for 15 min at RT. After this time, the absorbance was measured at 540 nm using a Synergy™ H1 reader (BioTek). The NO concentration in the trials was calculated based on the NaNO_2_ standard curve. The measurement of NO concentration using the Griess method was performed simultaneously with cytotoxicity analysis of crude latex (S1) and its fractions (S2, S3) against RAW 264.7 cells.

### 4.11. Enzyme-Linked Immunosorbent Assay (ELISA) for Investigating TNFα and IL-6 Secretion by RAW 264.7 Cells

In order to investigate how the crude *C. majus* latex and its fractions (alkaloid, protein) affect the secretion of tumor necrosis factor alpha (TNFα) and interleukin 6 (IL-6) by macrophages (RAW 264.7 cells), an enzyme-linked immunosorbent assay (ELISA) was used. A commercially available Tnf (mouse) ELISA Kit (Abnova, Taipei, China) and Il6 (mouse) ELISA Kit (Abnova) were used and the manufacturer’s instructions were followed. Controls were untreated cells, cells treated with 10% DMSO, and cells treated with LPS. An absorbance measurement for all samples at 450 nm (620 nm as reference wave) was performed using a Synergy™ H1 reader (BioTek). IL-6 or TNFα concentrations were determined from the standard curve. 

### 4.12. Plasmid DNA Isolation

Plasmid DNA from transformed Escherichia coli strain DH5α cells was isolated using the Plasmid Midi AX kit (A&A Biotechnology, Gdańsk, Poland). Isolation was performed according to the manufacturer’s instructions. After isolation, DNA concentration was measured spectrophotometrically using a BioTek Synergy™ H1 reader. The quality of the obtained DNA was checked by separating the samples on an agarose gel.

### 4.13. RNA Isolation

A commercially available Quick-RNA™ kit (Zymo Research, Irvine, CA, USA) was used to isolate total RNA from HeLa cells. RNA was isolated according to the manufacturer’s instructions.

### 4.14. Electrophoretic Separation of Nucleic Acids in Agarose Gel

Nucleic acids (RNA, DNA) were separated at a voltage of 150 V (10 V/cm) in a 1–1.5% agarose gel that was prepared from agarose dissolved in TAE buffer (40 mM Tris-HCl, pH 8.0; 20 mM acetic acid; 1 mM EDTA, pH 8.0) supplemented with 5 µL of SimplySafe™ dye (EurX) for every 100 mL of gel. Before loading onto the gel, a loading buffer (0.25% bromophenol blue, 0.25% xylene cyanol FF, 50% glycerol) was added to the samples in a 5:1 volume ratio. The size of the separated nucleic acid molecules was estimated based on the DNA size standards applied to the gel: the Perfect Plus™ 2 kb DNA Ladder (EurX) or Perfect Plus™ 1 kb DNA Ladder (EurX).

### 4.15. Reverse Transcription Real-Time PCR

The reverse transcription reaction was performed using the QuantiTect^®^ Reverse Transcription kit (QIAGEN, Hilden, Germany) according to the manufacturer’s instructions. A total of 300 ng of RNA was used for a single reverse transcription reaction. The cDNA was purified using the commercially available QIAquick PCR Purification Kit (QIAGEN) according to the manufacturer’s instructions. Real-time PCR was performed with the AmpliQ 5x HOT EvaGreen qPCR Mix Plus (no ROX) kit (Novazym) according to the manufacturer’s instructions. Primers used in the real-time PCR: E6-F:CCAGAAACCGTTGAATCCAG, R:GTTGGAGTCGTTCCTGTCGT; E7-F:GCGACTCAGAGGAAGAAAA, R:CAAAGGACAGGGTGTTCAG; GAPDH-F:AAGGTCGGAGTCAACGGATTT, R:ACCAGAGTTAAAAGCAGCCCTG. The reaction was performed in a Corbett Research Rotor-Gene 6000 thermocycler. The real-time PCR conditions were as follows: initial denaturation at 95 °C for 10 min; 40× (denaturation at 95 °C for 15 s, primer annealing at 50–54 °C for 20 s, elongation at 72 °C for 20 s); melting curve at 60–95 °C, increasing 1 °C every 5 s. The obtained results for the E6 and E7 genes were normalized to the results for the reference gene GAPDH (glyceraldehyde-3-phosphate dehydrogenase). Normalization was performed by dividing the obtained result by the value of the reference gene expression, thanks to which the relative levels of gene expression were obtained.

### 4.16. HPV Pseudoviron (PsV-HPV) Production in HEK293TT Cells

The p16sheLL (Addgene plasmid #37320) and pGL3-Basic (Promega) vectors used for the production of PsV-HPVs were obtained thanks to the courtesy of dr. Justyna Broniarczyk as a result of her stay at the Department of Tumour Virology at the International Centre for Genetic Engineering and Biotechnology (ICGEB) in Trieste, Italy. The vector p16sheLL contains two open reading frames encoding viral capsid proteins (L1, L2) [108]. The vector pGL3-Basic contains a luciferase-encoding reporter gene with which the PsV-HPV infection process can be monitored.

PsV-HPV16 pseudoviral particles were produced by transient transfection of eukaryotic cells with calcium phosphate. HEK293TT cells were transfected in 15 cm dishes (dish capacity: 25 mL of DMEM). The day before the transfection procedure, 3 × 10^6^ cells were loaded onto the dishes. For each vector, 4 dishes were seeded with cells. The next day, an appropriate amount of plasmid DNA of each vector was suspended in a CaCl_2_ solution in an Eppendorf tube, vigorously mixed, and then incubated for 20 min at room temperature. After 20 min, the HEBS solution (2×) was added to the samples and vigorously shaken to form DNA and calcium phosphate precipitates. The mixture prepared in this way was put on the plates with cells. The contents of the plates were mixed gently and then incubated for 48 h in an incubator (37 °C, 5% CO_2_). After this time, the medium was removed, the cells were washed with PBS solution (7–10 mL), and 2.5 mL of 0.05% trypsin solution was applied to each dish. The dishes were placed in the incubator for about 10–15 min or until the cells were detached from the bottom. For trypsin inactivation, 5 mL of fresh, warm DMEM was applied to all four dishes, which were collected together with the cells into a Falcon tube. Cells were centrifuged for 10 min at 4 °C and 3000 rpm (1700× *g*). After centrifugation, the supernatant was discarded and cells were resuspended in 1 mL of PBS solution and transferred to an Eppendorf tube. It was then centrifuged for 6 min at 8000 rpm (6200× *g*) at room temperature. After centrifugation, PBS was removed and a volume of 9.5 mM MgCl_2_ (relative to the volume of the cell pellet) was administered (e.g., 200 µL of MgCl_2_ was administered to 200 µL of the pellet) with gentle suspension. Then, depending on the volume of the cell pellet, after centrifugation, the Triton X-100 reagent (1/10 of the cell pellet volume), benzonase (0.4 μL/100 μL of cell pellet), and exonuclease V (0.4 μL/100 μL of cell pellet) were added. Everything was vigorously mixed and incubated for about 24 h at 37 °C with shaking at 300 rpm. The next day, a 0.17 volume (relative to the total sample) of cold 5 M NaCl was added to the samples (e.g., 72 µL of 5 M NaCl was applied to 421.6 µL). At the end of the test, it was mixed thoroughly and frozen at −80 °C until further use.

### 4.17. Purification of PsV-HPV16

Transfected cells were thawed at 37 °C for 5 min and then frozen again by transferring to −80 °C for 10 min to release PsV-HPV16 particles. This thaw–freeze cycle was repeated two more times. The tubes were then centrifuged for 6 min at 4 °C and 8000 rpm (6200× *g*). The supernatant was transferred to a silicone-coated Eppendorf tube, and the pellet was resuspended in 400 µL of HPV stabilization buffer (HSB) (125 mM HEPES, pH = 7.5; 2.5 M NaCl; 0.1% Brij58; 5 mM MgCl_2_; 500 μM EDTA; 2.5% ethanol) and centrifuged again. After centrifugation, the supernatant was transferred back to the silicone-coated tube, the pellet was resuspended in 400 µL of HSB (1×), centrifuged, and the supernatant was transferred. A two-step gradient of cesium chloride (CsCl) was then prepared by slowly feeding a 1.25 g/mL CsCl solution to the bottom of the centrifuge tube. By placing the serological pipette on the bottom of the tube, a 1.4 g/mL CsCl solution was carefully administered. Subsequently, pseudoviral particles suspended in HSB (1×) were applied from above. The sample prepared in this way was centrifuged with the balance tube in a pre-cooled SW 50.1 swing rotor (Beckman Coulter, Brea, CA, USA) in an Optima™ L-90K ultracentrifuge (Beckman Coulter, Brea, CA, USA) for 18 h at 20,000× *g* at 4 °C. After centrifugation, the tubes were transferred to a stand and left for 30 min at room temperature. The fraction (approx. 1.5 mL) containing the HPV pseudoviral particles was then withdrawn using a syringe with a needle and applied to an Amicon^®^ Ultra-4 100,000 kDa filter column (Merck). Then, 3 mL of HSB buffer (1×) was applied to the thus prepared column and centrifuged at 4 °C for 12 min at 4500 rpm (3900× *g*), and the filtrate was removed. To purify and concentrate PsV-HPV16 particles, the column was washed twice with HSB 1×. The suspension with HPV pseudoviral particles from the filter (approx. 50 μL) was then pipetted off and transferred to a silicone-coated Eppendorf tube. The concentration of pseudoviral particles in the obtained sample was measured spectrophotometrically by measuring the concentration of viral proteins (DeNovix DS-11 + spectrophotometer). The quality of the purified samples was checked by electrophoretic separation in a polyacrylamide gel under denaturing conditions and by staining with a Coomassie brilliant blue solution.

### 4.18. Electron Microscopy Observation of HPV Pseudovirions

PsV-HPVs were negatively stained on electron microscopy grids according to Brum and Steward, 2010. Electron microscopy to assess the quality of the produced HPV pseudoviral particles was carried out in the Laboratory of Electron and Confocal Microscopy at the Faculty of Biology, AMU. Images of the produced HPV pseudoviral particles (PsV-HPV) are provided in the Supplementary Materials (Figure S3).

### 4.19. Attachment Assay

The method for testing the attachment of PsV-HPV particles to HaCaT cells was performed according to Theisen et al., 2014 [96]. The technique is based on immunodetection of the viral capsid protein (L1). The level of L1 protein corresponds to the quantity of PsV-HPV particles attached to the cell surface. HaCaT cells were seeded in a 12-well plate at 60,000 cells per well and incubated overnight at 37 °C with 5% CO_2_. Then, the medium was removed and crude latex, appropriately diluted in DMEM, and dilutions of the tested fractions (alkaloid, protein) were administered and incubated for 1 h at 37 °C with 5% CO_2_. The controls were cells treated with 10% DMSO, cells not infected with virus, and cells untreated with crude latex and test fractions. Then, PsV-HPV particles were applied to the wells with cells at a concentration of 3 µg per well and incubated for 15 min at 4 °C with rocking. Subsequently, in order to remove PsV-HPVs not bound to the cell surface, the medium was removed and the cells were washed five times by administering and removing 1 mL of PBS solution each. On the fifth time, the PBS solution was removed and 50 µL of loading buffer (0.05 M Tris-Cl, pH = 6.8; 20% glycerol; 0.25% bromophenol blue; 4% SDS; 2% β-mercaptoethanol) was administered, in which cells were detached from the wells by means of sterile cell scrapers. Cells in SB buffer were harvested in 1.5 mL Eppendorf tubes and frozen at −20 °C until later use.

Subsequently, separation was performed on a 10% polyacrylamide gel under denaturing conditions and immunodetection of the L1 capsid protein was performed with an antibody recognizing the L1 protein (HPV16 L1: sc-47699, Santa Cruz Biotechnology). The secondary antibody concentration (sc-47699, Santa Cruz Biotechnology) was 1:400 and the loading control (loading control) was GAPDH. Then, the densitometric analysis was performed with the ImageJ software. 

### 4.20. PsV-HPV Infectivity Assay

The Luciferase Assay System (Promega) kit was used to analyze the effect of crude *C. majus* latex (S1) and the tested fractions (S2, S3) on the infectivity of PsV-HPV particles. This method is based on the presence of a reporter gene encoding luciferase in the PsV-HPV pseudogenome and the measurement of luminescence (light emission). The assay is based on the fact that when the pseudoviral particle enters the cell and pseudogenome reaches the nucleus, the luciferase gene is expressed and, consequently, luminescence is emitted. Hence, the level of luminescence corresponds to the amount of PsV-HPV particles that entered the cell and determines their infectivity. 

A total of 5000 HaCaT cells were seeded into each well of a 96-well plate and incubated overnight at 37 °C with 5% CO_2_. The medium was then removed and the crude C. majus latex diluted in DMEM or the diluted test fractions (S2, S3) was administered. PsV-HPV-untreated cells and cells not treated with any of the tested samples served as controls. One hour after the administration of crude latex and fractions, PsV-HPVs were administered to all wells with HaCaT cells, except the control, at a final concentration of 1 ng/μL, and the 96-well plate was incubated for 48 h at 37 °C at 5%. CO_2_. After this time, the DMEM was removed and the cells were washed with 100 µL of PBS buffer. Then, the PBS buffer was removed and 20 µL of lysis reagent (1×) was applied. Cell lysis was performed for 20 min at room temperature. After this time, 100 µL of an appropriately prepared luciferase substrate (Luciferase Assay Reagent) was administered and luminescence was measured using a Synergy™ H1 reader (BioTek). Wells with HaCaT cells untreated with PsV-HPVs were the “blank” sample. The read results were converted into relative PsV-HPV infectivity values expressed as a percentage according to the formula: (averaged measurement value for a given sample × averaged measurement value for a control)/averaged measurement value for a control × 100%. 

### 4.21. Molecular Docking of Selected C. majus Latex Alkaloids to p53 Protein

In order to analyze the possibility of the binding of *C. majus* latex alkaloids to the p53 protein, in silico molecular docking was carried out by the bioinformatics company Bioidea (Warsaw, Poland). The alkaloids were docked at three identified potential active sites of the p53 protein. The crystal structure of the wild-form p53 protein (PDB code: 2J0Z) was used, as were three alkaloids naturally occurring in C. majus latex: berberine (PubChem CID code: 2353), dihydroberberine (PubChem CID code: 10217), and coptisine (PubChem CID code: 72322).

### 4.22. Statistical Analysis

The results are plotted as the mean (with standard deviation) of three independent experiments (performed at least in two technical repetitions—duplicates). GraphPad Prism (version 5.01) was used to perform analyses and present the data in graphs. The statistical significance of differences between samples (means) was estimated using a one-way analysis of variance (ANOVA) with Tukey’s post hoc test. “*p*” values less than 0.05 were considered statistically significant.

## Figures and Tables

**Table 1:** Selected alkaloids identified by mass spectrometry in the *C. majus* latex alkaloid-rich fraction (S3).

Identification Results	RT (min)	[M]^+^ or [M + H]^+^	% (Semi-Quantitative)
coptisine	10.1	320.0912	9.30
chelidonine	9.0	354.1224	1.57
berberine	12.0	336.1225	3.43
stylopine	9.3	324.1229	1.35
protopine	8.2	354.1335	1.81

RT—retention time; [M]^+^ or [M + H]^+^—molecular ions; %—percentage content of alkaloids; all alkaloids identified by MS with their MS spectra are provided in Table S1.

**Table 2:** Selected proteins identified by mass spectrometry in the *C. majus* latex protein fraction. The table shows the results with score above 200 and arranged according to the decreasing score.

Identified Protein	Accession No. ^a^	Score ^b^	emPAI ^c^
polyphenol oxidase	m.60893	2703	4.16
reticuline oxidase-like	m.7838	2452	7.1
superoxide dismutase	uniq_01326	1764	5.62
major latex protein (MLP) 28	m.37901	426	2.07
peroxidase 12-like	m.7868	227	2.19
heat shock 70 kDa mitochondrial-like	m.61661	207	0.48

^a^ Accession numbers of the annotated transcript database of *C. majus* (version 2 Chmajus2015.01.06) [30] with added cRAP nucleotide sequences (common Random Protein Repository). The searchable *Chelidonium majus* coding sequence (CDS) database is provided on the IPK Gatersleben ViroBLAST server at: http://webblast.ipk-gatersleben.de/chelidonium/ (accessed on accessed on 24 January 2018). ^b^ Mascot search probability-based Mowse score. Ion score is −10 × Log(P), where P is the probability that the observed match is a random event. Score values of individual ions greater than 48 indicate the identity or very high homology of the match between the experimental data and the database sequence (*p* < 0.05). ^c^ Exponentially modified protein abundance index of identified protein according to Mascot search data, emPAI %—proportion of emPAI values in each category to the sum total of emPAI values for each sample (relative abundance). All proteins identified by MS are provided in Table S2.

**Table 3:** Comparison of the expression of E6 and E7 viral oncogenes at the mRNA and protein levels after treatment of HeLa cells with crude latex (S1) and its fractions (S2, S3). The table shows the percentage values for the expression level and the reduction in gene expression level compared to control (untreated cells). Both values add up to 100%.

Viral Oncogene		Crude Latex (S1)		Protein Fraction (S2)		Alkaloid-Rich Fraction (S3)		Untreated Cells (Control)	
		*mRNA*	*Protein*	*mRNA*	*Protein*	*mRNA*	*Protein*	*mRNA*	*Protein*
**E6**	Expression level ^a^	76%	23.7%	28%	67.9%	37%	75.5%	100%	100%
	Reduction in expression ^b^	24%	76.3%	72%	32.1%	63%	24.5%	0%	0%
**E7**	Expression level ^a^	80%	13.4%	24%	≥100%	37%	≥100%	100%	100%
	Reduction in expression ^b^	20%	86.6%	76%	0%	63%	0%	0%	0%

^a^ Gene expression level is a relative value based on the expression ratio of the analyzed genes (e.g., HPV E6, E7) versus a reference gene (GAPDH). The expression levels of analyzed genes from cells treated with the crude *C. majus* latex or one of its fractions (protein, alkaloid-rich) are compared with expression levels of genes in the control (untreated HeLa cells), the expression of which was always treated as 100%. ^b^ Reduction in expression is the value obtained by subtracting the expression values of the tested samples (e.g., HeLa cells treated with the crude latex) from the control. That is the reason why the relative expression level and reduction in expression values always add up to 100%.

**Table 4:** p53 protein and alkaloids selected for ligand–protein molecular docking.

Alkaloid/Protein	PDB Code/PubChem CID	Molecular Mass (g/moL)
p53	2J0Z	15,064.88
berberine	2353	336.367
dihydroberberine	10217	337.375
coptisine	72322	320.324

**Table 5:** The binding energy of ligand molecules at the active sites of the p53 protein.

Binding Energy (kcal/mol)	Berberine	Dihydroberberine	Coptisine
Active site 1	−6.7	−6.9	−7.4
Active site 2	−5.8	−5.7	−6.2
Active site 3	−5.8	−5.7	−6.3

## Data Availability

Data are contained within the article and Supplementary Materials.

## Supplementary Materials

The following supporting information can be downloaded at: https://www.mdpi.com/article/10.3390/ijms23169241/s1.
