# Supplementary material for: The Activity of Chelidonium majus L. Latex and Its Components on HPV Reveal Insights into the Antiviral Molecular Mechanism
Source: Int J Mol Sci. 2022 Aug 17;23(16):9241. doi: 10.3390/ijms23169241 (PMC9409487; doi:10.3390/ijms23169241)
Supplement: Supplementary file 1 [file ijms-23-09241-s001.zip › ijms-1866264-supplementary.pdf]

Supplementary Materials

Insights into the molecular mechanism behind *C. majus* latex antiviral properties. The activity of crude latex and its components on different stages of HPV infection.

Oskar Musidlak, Alicja Warowicka, Justyna Broniarczyk, Damian Adamczyk, Anna Goździcka-Józefiak and Robert Nawrot

Department of Molecular Virology, Institute of Experimental Biology, Faculty of Biology, Adam Mickiewicz University in Poznan, Uniwersytetu Poznanskiego 6, 61-614 Poznan, Poland;

1. *The verification of latex fraction composition*

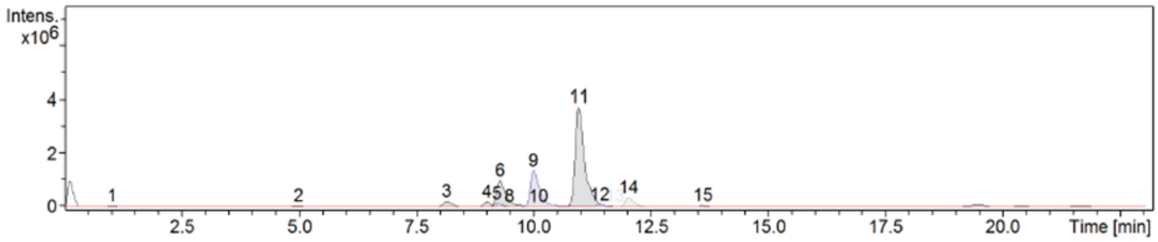

Figure S1. The total ion chromatogram and ESI mass spectra, taken form the RT range 1.0-13.6 min. Numbers 1-15 correspond to identified alkaloids in *C. majus* alkaloid-rich fraction (S3), depicted in Table S1.

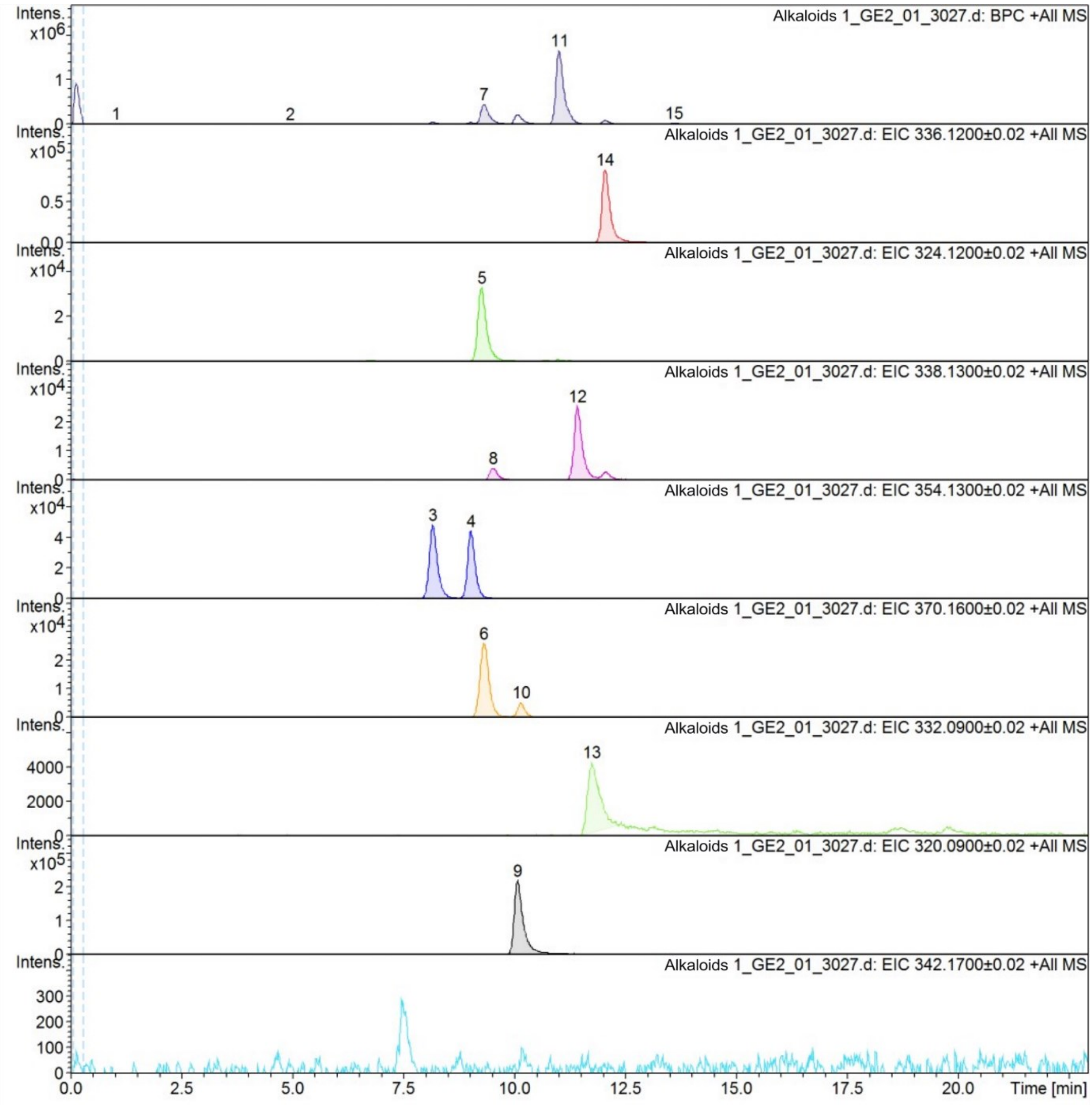

Figure S2. Extracted ion chromatograms list from HPLC-MS analysis of *C. majus* latex alkaloid-rich fraction. Numbers 1-15 correspond to identified alkaloids in *C. majus* alkaloid-rich fraction (S3), depicted in Table S1.

Table S1. Alkaloids identified by mass spectrometry in the *C. majus* latex alkaloid-rich fraction (S3).

No.	RT	$[M]^+$ or $[M+H]^+$	Identification results	Alkaloid fraction (#1)	Alkaloid fraction (#2)
	[min]	m/z		% (semi-quantitative)	
**1**	1.0	115.0364	unknown	0.34	0.39
**2**	4.9 – 5.0	350.1020	unknown	0.07	0.38
**3**	8.2	354.1335	protopine	1.81	2.48
**4**	9.0	354.1224	chelidonine	1.57	1.94
**5**	9.3	324.1229	stylopine	1.35	1.40
**6**	9.3	370.1649	allocryptopine isomer	1.13	1.37
**7**	9.3	322.1071	unknown	17.83	13.55
**8**	9.5	338.1379	jatrorrhizine/isomer	0.16	0.19
**9**	10.1	320.0912	coptisine	9.30	18.94
**10**	10.1	370.1649	allocryptopine	0.18	0.19
**11**	11.0	380.1493	unknown		
**12**	11.4	338.1383	dihydroberberine	0.98	0.92
**13**	11.7	332.0923	sanguinarine	0.23	0.25
**14**	12.0	336.1225	berberine	3.43	4.42
**15**	13.6	348.1228	chelerythrine	0.82	0.38
**n.a.**	0.1	226.9517	- (solvents+additives)		

RT – retention time; $[M]^+$ or $[M+H]^+$ - molecular ions; % - percentage content of alkaloids. Semi-quantitative analysis was performed in duplicate (#1, #2).

Table S2. Proteins identified by mass spectrometry in the *C. majus* latex protein fraction (S2). The table shows the results with score above 200 and arranged according to the decreasing score.

Accession No.[a)]	Identification results	Score[b)]	MW [Da][c)]	Peptide count[d)]	Sequence coverage[e)]	emPAI[f)]
m.61435	ubiquitin-nedd8-like protein rub1-like	4975	17103	9	54	10,32
m.60893	polyphenol oxidase	2703	66876	22	37	4,16
m.7838	reticuline oxidase-like	2452	62494	24	48	7,1
uniq_01326	superoxide dismutase	1764	15292	7	42	5,62
m.2906	polyphenol chloroplastic-like	1181	67955	18	37	2,46
m.54853	beta-amylase precursor	1167	57143	11	26	2,02
m.60799	lactoylglutathione lyase-like	1055	38664	12	34	4,7
m.60938	phospholipase d alpha 1-like	814	92993	18	32	1,48
m.44308	reticuline oxidase-like	652	59389	6	12	0,76
m.62963	polyphenol chloroplastic-like	590	57726	8	20	1,08
m.61212	malate mitochondrial-like	530	37227	8	28	1,47

m.61052	superoxide dismutase	507	21484	5	35	1,63
m.44293	reticuline oxidase	471	43115	6	16	1,18
m.37901	mlp-like protein 28	426	41201	10	25	2,07
m.37898	mlp-like protein 28	415	39557	8	23	1,6
m.2548	enolase-like	412	47955	6	18	0,69
m.24014	calmodulin-like isoform 1	391	16883	8	53	10,68
m.60753	calmodulin-related protein isoform 1	390	24274	9	55	5,62
m.7868	m.7868 peroxidase 12-like	388	25142	7	32	2,19
m.16756	phosphoglycerate cytosolic-like	380	42300	10	35	1,7
m.60989	protein disulfide-isomerase-like	370	56569	10	22	1,11
m.2283	acid beta-fructofuranosidase-like	369	58014	5	14	0,44
m.12634	mlp-like protein 28	340	21820	8	41	3,6
m.14064	protein in2-1 homolog b-like	340	33956	6	31	1,1
m.62107	malate chloroplastic-like	338	43545	6	18	0,79
m.43933	atp synthase subunit mitochondrial-like	336	59908	7	17	0,64
m.60714	glyceraldehyde-3-phosphate cytosolic-like	314	36928	5	16	0,76
m.17988	glyceraldehyde-3-phosphate cytosolic-like	309	46280	9	33	1,27
m.2644	polyphenol oxidase	308	49158	5	10	0,67
m.61561	DNA repair protein rad23-3	291	41475	6	19	1,03
m.3150	minor allergen alt	284	21592	5	32	1,62
m.2591	monodehydroascorbate reductase-like	281	35425	6	19	1,04
m.16751	desiccation protectant protein lea14 homolog	279	19103	5	31	1,97
m.2541	anthocyanidin -o-glucosyltransferase-like	274	49082	9	22	1,16
m.59541	protein disulfide isomerase-like 1-4-like isoform 1	266	65116	7	12	0,57
uniq_02123	s-norcoclaurine synthase-like	265	18108	6	55	2,96

m.11641	stromal 70 kda heat shock-related chloroplastic-like	262	76045	7	11	0,48
m.61381	glutathione cytosolic-like	241	54377	6	16	0,59
m.60932	luminal-binding protein 5-like	238	73905	7	12	0,49
m.60758	malate cytoplasmic-like	238	41542	4	12	0,66
uniq_03599	probable calcium-binding protein cml13-like	229	16548	5	38	3,5
m.2578	peroxidase 12-like	227	39347	7	19	1,35
m.61081	multiprotein-bridging factor 1b-like	218	15623	5	35	2,75
m.61661	heat shock 70 kda mitochondrial-like	207	72835	5	9	0,48
m.33249	ribulose-phosphate 3-cytoplasmic isoform-like	205	24303	3	16	0,99
m.12629	mlp-like protein 34	203	11788	5	42	7,12

a) Accession numbers of the annotated transcript database of *C. majus* (version 2 Chmajus2015.01.06) with added cRAP nucleotide sequences (common Random Protein Repository). The Searchable *Chelidonium majus* coding sequences (CDS) database is provided on IPK Gatersleben ViroBLAST server at: http://webblast.ipk-gatersleben.de/chelidonium/

b) Mascot Search Probability Based Mowse Score. Ions score is −10∗Log(P), where P is the probability that the observed match is a random event. Score values of individual ions greater than 48 indicate the identity or very high homology of the match between the experimental date and the database sequence ($P < 0.05$).

c) Theoretical molecular weights (kDa) of the identified proteins.

d) Number of "matching" peptides identified by searching databases with the MASCOT algorithm.

e) The degree of coverage of the amino acid sequences of the identified proteins

f) Exponentially modified protein abundance index of identified protein according to Mascot Search data emPAI % — proportion of emPAI values in each category to the sum total of emPAI values for each sample (relative abundance).

2. *In silico docking of selected C. majus latex alkaloids to p53 protein*

The identified coordinates (x, y, z) of active sites were used for the docking of ligands (berberine, dihydrobberine and coptisine) to p53. The first active pocket was identified by 6 predictors and is the highest-rated active site in the p53 protein (Table S3).

Table S3. Identification of coordinates (x, y, z) of p53 active sites.

Active site No.	x	y	z	Number of predictors	z-score
1	-2.082	1.907	7.032	6	16.08
2	3.406	-7.713	3.014	1	3.43
3	14.191	-2.847	4.276	2	2.80

MetaPocket software also determined the amino acid residues within the active pocket. These are the rest that can potentially bind to ligand molecules. The list of amino acid residues making up the active pockets is provided in Table S4.

Table S4. Amino acid residues identified in acitve sites of p53.

**Active site 1**									
GLU_A^349^	ARG_B^333^	ARG_B^337^	ASP_A^352^	GLY_B^334^	ARG_B^335^	GLU_B^336^	GLU_B^339^	MET_B^340^	GLU_B^343^
GLU_D^336^	LEU_A^348^	ARG_B^342^	LYS_C^351^	ARG_D^337^	MET_D^340^	LEU_B^344^	LEU_C^348^	ALA_A^353^	LYS_A^351^
LEU_A^350^	ALA_A^355^	ALA_A^347^	GLU_D^339^	GLU_D^343^	ARG_D^335^	GLY_D^334^	ARG_D^342^	LEU_A^344^	LEU_D^344^
GLU_A^343^	ALA_D^347^	LEU_D^348^	GLU_D^346^						
**Active site 2**									
GLN_A^354^	ALA_A^355^	LYS_A^351^	LEU_D^350^	GLU_D^343^	GLU_D^346^	ALA_D^347^	ALA_A^347^	LEU_A^348^	LEU_A^350^
GLN_D^354^	GLU_A^346^	LYS_D^351^	ALA_D^353^	MET_B^340^	GLU_D^339^	MET_D^340^	LEU_A^344^	LEU_D^344^	GLU_A^339^
MET_A^340^	GLU_A^343^	MET_C^340^	ARG_C^337^	LEU_D^348^	GLU_A^336^	GLU_C^343^	GLU_C^336^	ARG_A^335^	ARG_A^337^
GLU_C^339^	ARG_A^342^								
**Active site 3**									
ASP_C^352^	ARG_D^333^	ASN_C^345^	LEU_C^348^	GLU_C^349^	ILE_D^332^	GLY_D^334^	GLN_D^331^	LYS_C^351^	GLN_C^331^
ASN_D^345^	GLU_D^349^								

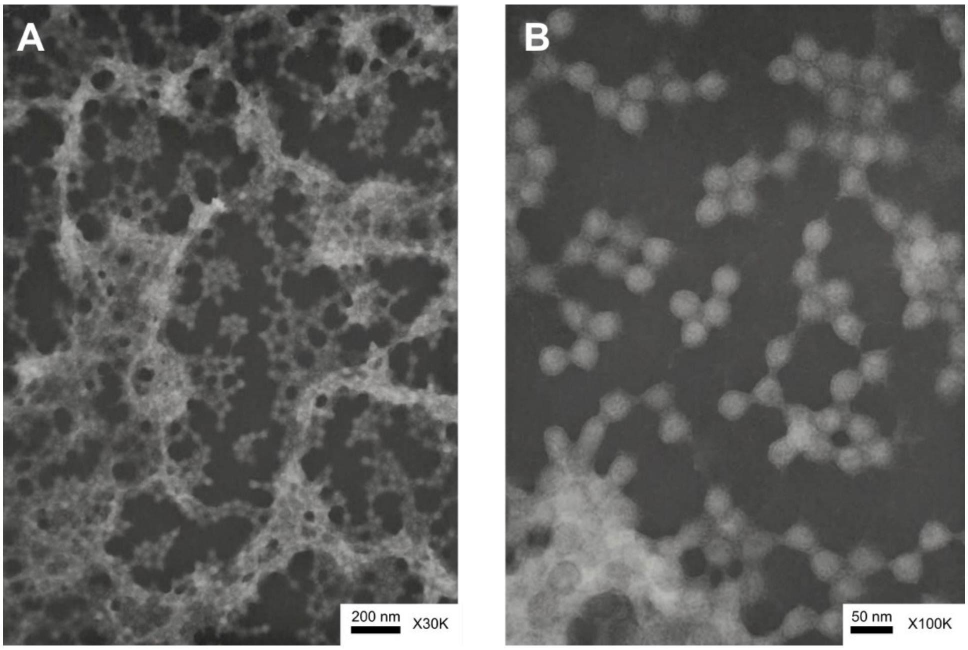

Figure S3. Images of HPV pseudoviral particles (PsV-HPV) produced in HEK293TT cells. Micrographs obtained using Transmission Electron Microscope at 30 000X magnification, scale bar = 50 nm (A) and 100 000X magnification, scale bar = 50 nm (B).
